# Expanding the *β*‐Lactamase Family in the Human Microbiome

**DOI:** 10.1002/advs.202403563

**Published:** 2024-10-24

**Authors:** Baolei Jia, Ju Hye Baek, Jae Kyeong Lee, Ying Sun, Kyung Hyun Kim, Ji Young Jung, Che Ok Jeon

**Affiliations:** ^1^ Xianghu Laboratory Hangzhou 311231 China; ^2^ Department of Life Science Chung‐Ang University Seoul 06974 Republic of Korea; ^3^ Department of Veterinary and Animal Sciences University of Copenhagen Copenhagen 1870 Denmark; ^4^ Department of Biological Sciences and Biotechnology Hannam University Daejon 34054 Republic of Korea; ^5^ Microbial Research Department Nakdonggang National Institute of Biological Resources Gyeongsangbuk‐do 37242 Republic of Korea

**Keywords:** antibiotic resistance, antibiotic stewardship, *β*‐Lactams, global *β*‐lactamase profiling, protein family

## Abstract

*β*‐lactams, the most common antibiotics globally, have resistance primarily determined by *β*‐lactamases. Human microbiota and *β*‐lactams influence mutually; however, *β*‐lactamase variety and abundance in the human microbiome remain partially understood. This study aimed to elucidate the diversity, abundance, and substrate spectrum of *β*‐lactamases. 1369 characterized *β*‐lactamases and 16 204 putative sequences are collected from protein databases. Upon clustering analysis and biochemical assays, nine proteins exhibiting less than 35% identity to those previously characterized are confirmed as *β*‐lactamases. These newly identified *β*‐lactamases originated from eight distinct clusters comprising 1163 *β*‐lactamases. Quantifying healthy participants (n = 2394) across 19 countries using functionally confirmed clusters revealed that Japan have the highest gut *β‐lactamase* abundance (log_2_[reads per million (RPM)] = 6.52) and Fiji have the lowest (log_2_[RPM] = 2.31). The *β‐lactamase* abundance is correlated with *β*‐lactam consumption (R = 0.50, *p* = 0.029) and income (R = 0.51, *p* = 0.024). Comparing individuals with ailments with healthy participants, *β‐lactamase* abundance in the gut is increased significantly in patients with colorectal cancer, cardiovascular diseases, breast cancer, and epilepsy. These outcomes provide insights into investigating antibiotic resistance, antibiotic stewardship, and gut microbiome‐antibiotic interactions.

## Graphical abstract

*β*‐lactam resistance is mainly driven by *β*‐lactamases. The study analyzes 1369 characterized and 16 204 putative *β*‐lactamases, identifying nine from eight clusters. The highest gut *β*‐lactamase abundance is found in Japan and the lowest in Fiji. *β*‐lactamase levels correlate with *β*‐lactam use and income, increasing in colorectal cancer and cardiovascular disease patients. These outcomes offer insights into antibiotic stewardship.

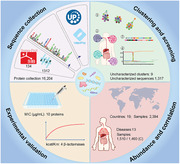

## 1 Introduction

The widespread use of antibiotics, which began in the 1940s to prevent and treat bacterial infections, has been a successful chemotherapy form in the 20th century, saving many lives.^[^ ^1^ ^]^ However, in 2019, 13.6% (13.7 million) of global deaths were associated with bacteria, making it ranked the second leading cause of mortality globally, following ischemic heart disease. The contribution of bacterial resistance to antimicrobials (AMR) is significant in these deaths, as ≈4.95 million fatalities in 2019 were linked to bacterial AMR, with 1.27 million of them directly associated with AMR.^[^ ^2^ ^]^ Among the antibiotics, the *β*‐lactam class was first recognized upon the discovery of penicillin and has become the most used and successful antibiotic class for treating infections.^[^ ^3^ ^]^ However, the use of *β*‐lactam antibiotics usually causes the emergence of AMR.

The *β*‐lactam class of antibiotics is characterized by a four‐membered cyclic amide core structure. Resistance mechanisms to *β*‐lactams involve limiting access to DD‐transpeptidases, reducing their binding affinity, and degrading the antibiotic using *β*‐lactamases.^[^ ^4^ ^]^ *β*‐lactamases degrade antibiotics by breaking the amide bond within the *β*‐lactam ring. These enzymes, produced by both Gram‐positive and Gram‐negative pathogens, are the most significant resistance mechanism. Hence, elucidating the structure, catalysis, and mechanism of *β*‐lactamases to design an inhibitor for the enzymes is particularly attractive to the clinical field.^[^ ^5^ ^]^ *β*‐lactamases are highly diverse and exhibit promiscuous activity. They are categorized into Ambler classes A, B, C, and D based on protein sequence similarity. Classes A, C, and D have a serine active site in the conserved Ser‐X‐X‐Lys motif, while class B *β*‐lactamases use Zn^2+^ for hydrolysis.^[^ ^6^ ^]^ In the clinical setting, penicillinase, cephalosporinase, inhibitor‐resistant *β*‐lactamases, and extended‐spectrum *β*‐lactamases are common designations for these enzymes. The continuous evolution of *β*‐lactamases, driven by antibiotic pressure, increases their specificity and efficiency, complicating the development of effective inhibitors.^[^ ^7^ ^]^

**Figure 1:**
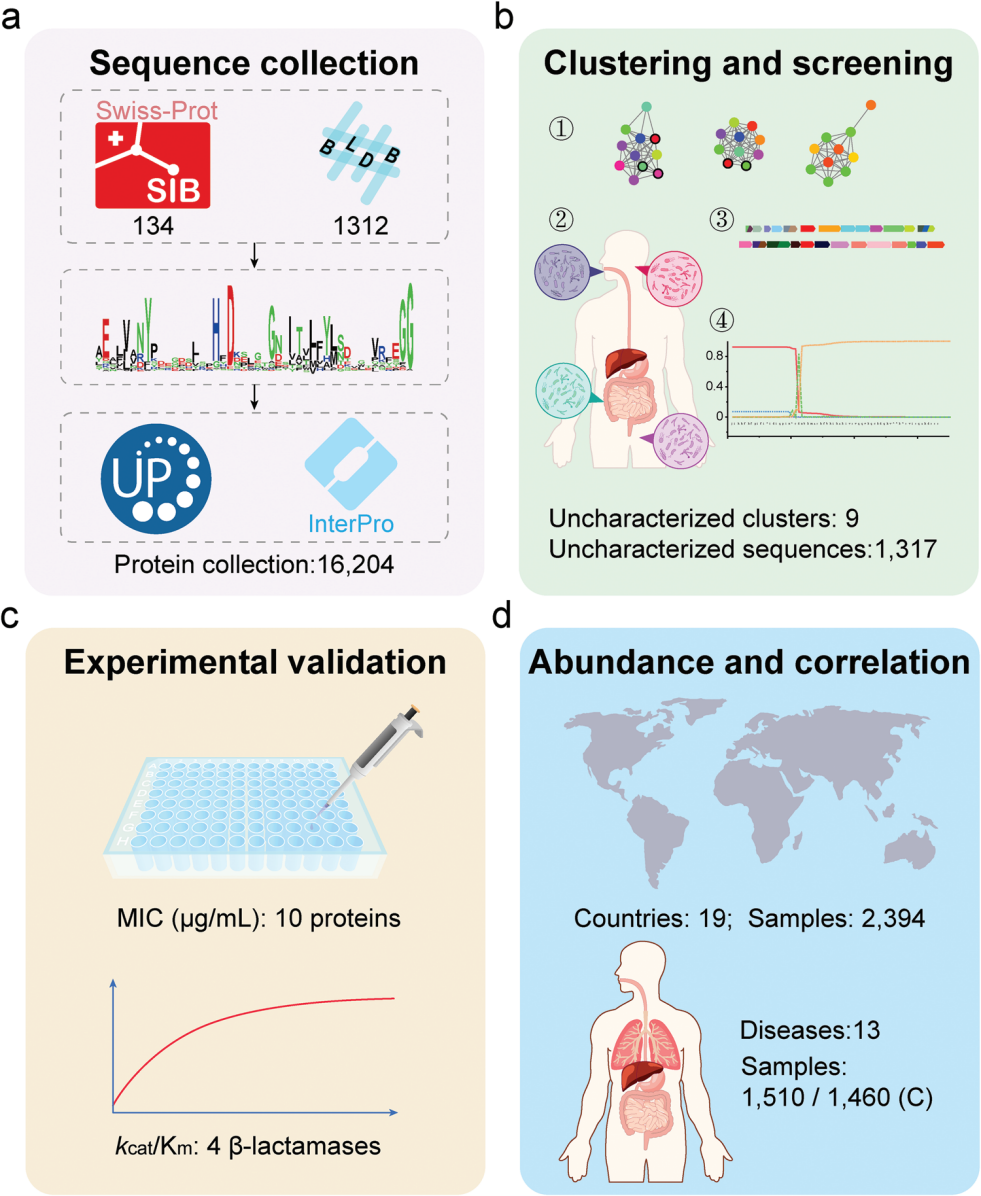
Schematic diagram of research workflow. a) Characterized *β*‐lactamase sequences were collected from the Swiss‐Prot database and Beta‐Lactamase DataBase (BLDB). Hidden Markov models (HMMs) for each class were constructed to mine potential *β*‐lactamases in UniProt and InterPro databases. b) The potential *β*‐lactamases were selected using four steps: 1) protein sequence similarity network, 2) isolation origin from the human microbiome, 3) genomic context analysis, and 4) signal peptides screening. c) Promising candidates were screened by detecting the minimum inhibitory concentration (MIC) and activity of the enzymes and verified by experiments. d) The relationship between the abundance of *β*‐lactamases, antibiotic consumption in different countries, and different diseases was also analyzed.

Addressing the challenges posed by *β*‐lactamases is essential for achieving the United Nations Sustainable Development Goal 3.8, which aims toward “access to safe, effective, quality, and affordable essential medicines and vaccines for all.”^[^ ^8^ ^]^ A deeper understanding of the diversity and abundance of *β*‐lactamases can contribute to achieving the goal. Moreover, the widespread nature of antibiotic resistance emphasizes the need to monitor potential *β*‐lactamase hotspots for emerging resistance threats before they spread. This includes cryptic *β*‐lactamases, which reside within a bacterial chromosome but are not evidently linked to antibiotic resistance.^[^ ^9^ ^]^ The development of metagenomic techniques has been a crucial driver in *β*‐lactamase studies and has revealed that the genes encoding *β*‐lactamases are abundant in the soil,^[^ ^10^ ^]^ river,^[^ ^11^ ^]^ sewage,^[^ ^12^ ^]^ and animal,^[^ ^13^ ^]^ microbiomes. The human microbiome, particularly those of the oral and gut, are also reservoirs of genes encoding *β*‐lactamases.^[^ ^14a,b^ ^]^ As a *β*‐lactamase source, the human microbiome can easily contribute to their spread between countries.^[^ ^15a,b^ ^]^ Colonization of *β*‐lactamase‐producing *Enterobacteriaceae* may increase ulcerative colitis (UC) disease severity.^[^ ^16^ ^]^ Furthermore, fecal microbiota transplantation with *β*‐lactamase‐producing *E. coli* may cause bacteremia.^[^ ^17^ ^]^ Hence, elucidating the diversity, abundance, and substrate spectrum of *β*‐lactamases is crucial. In this study, We aimed to identify potential *β*‐lactamases and the microbes harboring these enzymes within the human microbiome using a four‐tiered approach, including 1) ingathering of *β*‐lactamase sequences from an expert *β*‐lactamase database and the Universal Protein databases; 2) mining of novel *β*‐lactamases using sequence similarity networks and genome context analysis in the human microbiome; 3) the use of microbiological and biochemical experiments to analyze the spectrum of the enzymes and inhibition of the microorganisms by antibiotics; 4) the use of the confirmed *β*‐lactamase sequences to interpret the relationship between the abundance of the genes in the human gut microbiome, *β*‐lactam consumption, and disease conditions (**Figure** **1**).

## 2 Results

### 2.1 Identification of Potential *β*‐Lactamases Within Publicly Available Databases

**Figure 2:**
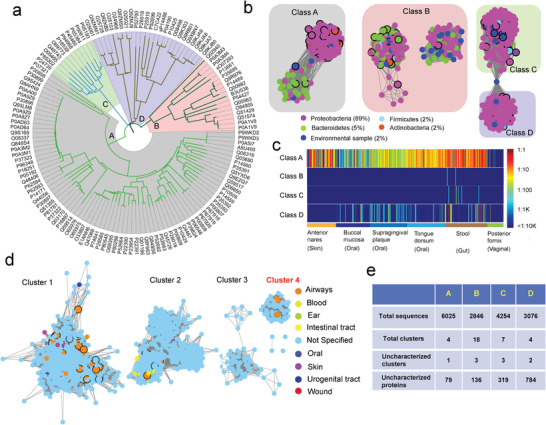
Establishing the prediction pipeline of *β*‐lactamases. a) The retrieval of 134 experimentally characterized *β*‐lactamases from the Swiss‐Prot database and the phylogenetic trees showing that these proteins could be classified into A, B, C, and D classes. b) The use of a protein sequence similarity network using a 40% sequence identity cut‐off to separate the 134 and 1312 known *β*‐lactamases from the BLDB. The proteins from the Swiss‐Prot database are enlarged and labeled with a black circle. Proteins from the same phylum are labeled with the same color, and the percentage from the phylum is listed at the bottom right. c) Heatmap depicting the abundance and distribution of the four *β*‐lactamase classes across six body sites, as determined through ShortBRED analysis. d) Using a 40% sequence identity to divide 6025 homologs of Class A *β*‐lactamases into four clusters in the SSN. The homolog proteins were obtained by searching the protein databases using the known proteins identified in Figure 2b. The proteins originating from the human microbiome of different body sites are represented in a different color and enlarged. The color corresponding to the body sites is shown to the right. e) Summary of the total homolog protein sequences, total clusters, uncharacterized clusters, and uncharacterized proteins in each class of *β*‐lactamases.

We analyzed the characterized *β*‐lactamases from previous studies of two resources (Swiss‐Prot database and Beta‐Lactamase DataBase [BLDB]). The Swiss‐Prot database contains protein sequences with accurate detailed annotations,^[^ ^18^ ^]^ and the BLDB is a manually curated database of the structure and function of *β*‐lactamases.^[^ ^19^ ^]^ We searched the Swiss‐Prot database using “EC 3.5.2.6” and obtained 134 experimentally characterized *β*‐lactamases (**Figure** **2**a; Dataset S1, Supporting Information). Phylogenetic analysis showed that the characterized proteins from the Swiss‐Prot database could be grouped into four classes, namely A, B, C, and D; however, half of the characterized *β*‐lactamases belonged to Class A. We further analyzed the BLDB database, which embodied 7739 sequences. According to the PubMed ID in the database, we obtained 1312 *β*‐lactamases (Dataset S1, Supporting Information), which could be assigned to Classes A (585), B (196), C (214), and D (317). Once we combined the protein sequences from the two resources and removed those that were redundant, we obtained 1369 protein sequences, which were used to build the protein sequence similarity network (SSN) using a 40% sequence similarity cut‐off. The visualization showed that Class B could be further grouped into two clusters in the network, and the grouping would have remained if the network were constructed using a 20% sequence similarity cut‐off (Figure S1, Supporting Information), implying that the proteins from the two clusters showed excessively low sequence identity and that convergent evolution at the functional level might occur for Class B *β*‐lactamases. In contrast, *β*‐lactamases from Classes C and D were associated with proteins (B5L5V6) originally from remote Alaskan soil,^[^ ^20^ ^]^ suggesting that the *β*‐lactamases from the two classes may be evolutionally derived from the same source. Among the 1369 proteins, 1327 had signal peptides (Figure S2, Supporting Information), accounting for 96.9% of the total experimentally characterized *β*‐lactamases. Moreover, these characterized enzymes were primarily from Proteobacteria (89%). Only a few *β*‐lactamases were from Bacteroidetes (5%), Firmicutes (2%), Actinobacteria (2%), and Environmental samples that cannot be taxonomically assigned at the phylum level (2%). The predominance of characterized enzymes from Proteobacteria is likely due to the intensive research focus on antibiotic resistance within this phylum. Many species within Proteobacteria, such as *Escherichia coli*, *Pseudomonas aeruginosa*, *Klebsiella pneumoniae*, *Legionella pneumophila*, and *Salmonella* species, are well‐known for their significant roles in *β*‐lactam studies.

We used ShortBRED to examine the occurrence of four *β*‐lactamase classes within 380 metagenomic datasets (Dataset S2, Supporting Information) obtained from healthy participants during the HMP (Figure 2c). These metagenomic samples were collected from six human body sites, namely anterior nares (facial skin), oral cavity (buccal mucosa, supragingival plaque, and tongue dorsum), fecal samples, and vaginal fornix. All four *β*‐lactamase classes were identified in the human microbiome through their SSN sequences; however, their prevalence and distribution varied significantly. Class A enzymes were the most abundant across the six body sites (Figure 2b). Class D *β*‐lactamases were also in oral, fecal, and posterior vaginal samples, whereas Classes B and C enzymes exhibited relatively low abundance and limited distribution in human microbiomes.

We built the profiles of the *β*‐lactamases for each Class using the hidden Markov model (HMM, Figure S3 and Dataset S3, Supporting Information) based on the characterized *β*‐lactamases from a previous study. The profiles were used to search the homologs of *β*‐lactamases from the UniProt and InterPro databases, which are universal protein databases containing many uncharacterized and novel proteins. Finally, 6025, 2846, 4254, and 3076 protein sequences belonging to Classes A, B, C, and D, respectively (Dataset S4, Supporting Information), were obtained from the databases.

Subsequently, four steps using 4 criteria were employed to screen the novel *β*‐lactamases. These criteria included: protein sequence similarity of less than 40% with characterized proteins, isolation origin from the human microbiome, genomic context associated with lactam metabolism, and the presence of N‐terminal signal peptides. We used the Class A *β*‐lactamases to explain the steps for screening the novel proteins in this family. First, the protein SSNs were built based on the sequences using a 40% sequence similarity cut‐off (Figure 2d). The 6025 protein sequences were grouped into four clusters, and 4883, 973, 86, and 79 sequences belonged to Clusters 1, 2, 3, and 4, respectively. Among them, Clusters 1 and 2 contained the characterized *β*‐lactamases, implying that their proteins may share similar biochemical properties as those of the characterized enzymes. No proteins in Clusters 3 and 4 showed high similarities to the characterized *β*‐lactamases, suggesting that these proteins may represent novel *β*‐lactamases that have not been studied. Hence, the low sequence similarity was set as a criterion to screen novel *β*‐lactamases. Second, microorganisms isolated from human‐associated environments (e.g., respiratory tract, skin, intestinal tract, oral cavity) are more likely to be clinically significant, as these are common sites of infection. This was established as the second criterion for selection. Since some sequences from Clusters 1 and 2 were from microorganisms isolated from these human‐associated sites, these clusters were included for further analysis. However, no sequences in Cluster 3 were from the human microbiome, leading to its exclusion. Regarding Cluster 4, two proteins were from *Pseudomonas stutzeri*, which is an opportunistic pathogen occupying diverse ecological niches, including the human respiratory tract and skin, suggesting that the proteins in Cluster 4 prioritize human health. Third, when we analyzed the genome context of each cluster using the genome neighborhood network (GNN) (Figure S4, Supporting Information), the data showed that Cluster 4 proteins may be located with genes encoding penicillin‐binding protein, suggesting that these proteins showed a high possibility of having lactam metabolism functions. Similarly, the genes encoding penicillin acylase,^[^ ^21^ ^]^ alkyl sulfatase,^[^ ^22^ ^]^ and histidine kinase^[^ ^23^ ^]^ have been shown to be located near antibiotic‐degrading *β*‐lactamases in the genome. Thus, the genomic context of the proteins in the clusters was used as the third criterion to filter the sequences. Next, the occurrence of signal peptides at the N‐terminus was used as the fourth criterion because 96.9% of the characterized *β*‐lactamases have these peptides, and Cluster 4 proteins also met this standard. We set four criteria to screen the uncharacterized Class A *β*‐lactamases, and one cluster in this class containing 79 sequences was finally selected for further confirmation.

Based on the same procedure, we divided the sequences in Classes B, C, and D into 18, 7, and 4 clusters, respectively (Figures S5–S7, Supporting Information). After screening, Clusters 9, 12, and 17 containing 80, 41, and 15 sequences (136 sequences in total), respectively, were identified as uncharacterized enzymes in the human microbiome in the Class B *β*‐lactamases. In the Class C *β*‐lactamases, Clusters 2, 3, and 4 with 162, 104, and 52 sequences (319 sequences in total), respectively, were proposed as putative *β*‐lactamases. Furthermore, Clusters 3 and 4, containing 630 and 154 sequences (784 sequences in total), respectively, in Class D, were screened for further analysis (Figure 2e). By integrating the SSN results and genomic context, we expanded the *β*‐lactamase pools extensively for further analysis.

### 2.2 Experimental Validation Substantiates the Activity of the Screened *β*‐Lactamases

**Figure 3:**
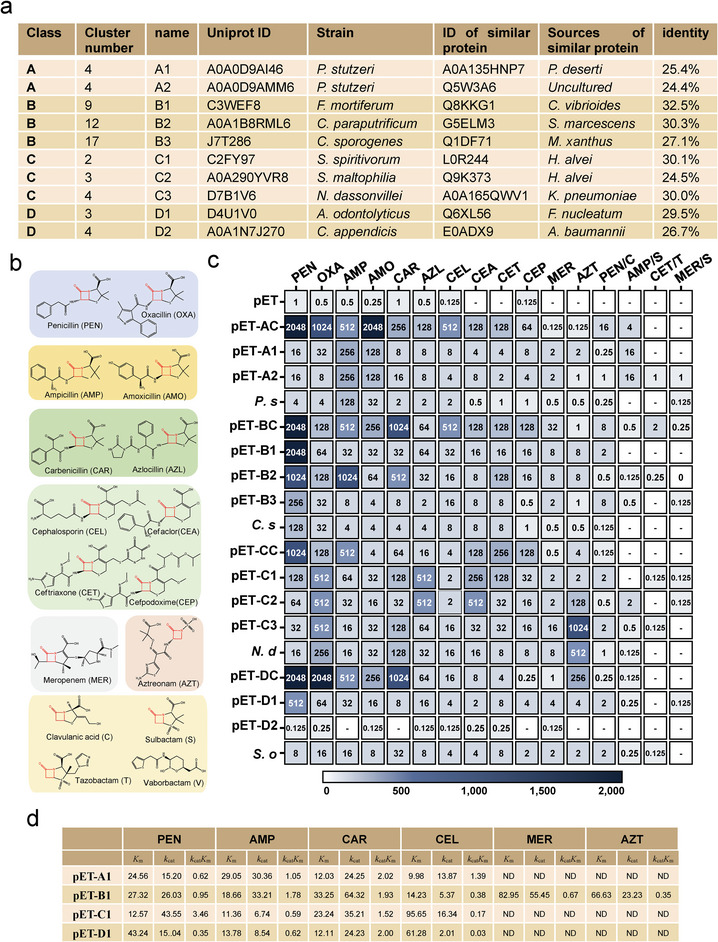
Experimental validation of the predicted *β*‐lactamases. a) Summary of the selected proteins for experimental validation. We selected 10 proteins from different clusters of each class of *β*‐lactamases and named them sequentially. The bacteria harboring the protein, the origin of the bacteria, and the known enzymes with the highest sequence identity to the selected proteins are shown. b) The lactam antibiotics used as substrates for experimental validation. The narrow‐spectrum, broad‐spectrum, extended‐spectrum, and cephem lactams, as well as the carbapenem, monobactam, and *β*‐lactamase inhibitors, are listed from top to bottom and shaded in different colors. c) The minimum inhibitory concentration (MIC) range of the selected *β*‐lactamases and the known *β*‐lactamases against the *β*‐lactams. The MIC range of the selected *β*‐lactamases and the known *β*‐lactamases against the *β*‐lactams. *P. s*, *C. s*, *N. d*, and *S. o* represent the four strains of *Pseudomonas stutzeri*, *Clostridium sporogenes*, *Nocardiopsis dassonvillei*, and *Schaalia odontolytica*, respectively. “‐” means the MIC value was not determined. d, Kinetic parameters of the A1, B1, C1, and D1 enzymes to different antibiotics.

Sequence alignments using a cut‐off threshold of 40% effectively differentiate between proteins with similar and dissimilar functions.^[^ ^24^ ^]^ This implies that one or two proteins can adequately represent the functional characteristics of the entire protein group within a cluster. To verify the function of the putative *β*‐lactamases in the new clusters, we selected two sequences in Cluster 4 of the Class A *β*‐lactamases, as the two proteins were from *P. stutzeri* and occupy one locus on the genome. In the other clusters of Class B, C, and *β*‐lactamases, one sequence was selected from each cluster and named sequentially. The sequences were selected from the human microbiota, as these bacteria have more potential to be related to human health. For example, *Fusobacterium mortiferum* encoding B1 in Class B is common in the human mouth and gastrointestinal tract. This strain has been associated with periodontal disease and infections in other body parts, such as the brain and liver.^[^ ^25^ ^]^ Subsequently, we selected 10 putative *β*‐lactamases for biochemical characterization and verification (**Figure** **3**a). The highest sequence identity between the 10 selected proteins and characterized *β*‐lactamases was 32.5% (B1 to a *β*‐lactamase from *Caulobacter vibrioides*), and the lowest identity was 24.4% (A2 to a *β*‐lactamase from uncultured bacteria). Therefore, our selected putative enzymes had low sequence similarity to known *β*‐lactamases. We further selected four characterized *β*‐lactamases from each class as the controls and named them AC (Class A for Control), BC, CC, and DC (Table S1, Supporting Information). Next, we optimized the DNA sequences of the 14 proteins for expression in *E. coli* (Dataset S5, Supporting Information). The optimized sequences were synthesized and cloned into a pET26 vector for expression. During cloning, the sequences encoding the signal peptide of the *β*‐lactamases were removed, and the signal peptide sequence in the pET26 vector was used to render a consistent secretion for each protein. After transformation and screening, all 14 proteins were sufficiently expressed in *E. coli* (Figure S8, Supporting Information).

Furthermore, we examined the antibiotic resistance activity of the 14 proteins by assessing the minimal inhibitory concentration (MIC, µg mL^−1^) of various antibiotics against the transformants (Figure 3b,c). These antibiotics included narrow‐spectrum lactams (penicillin G and oxacillin), broad‐spectrum lactams (ampicillin and amoxicillin), extended‐spectrum lactams (carbenicillin and azlocillin), first‐ to third‐generation cephems (cephalosporin, cefaclor, ceftriaxone, and cefpodoxime), carbapenems (meropenem), and monobactams (aztreonam). A combination of four *β*‐lactamase inhibitors (clavulanic acid, sulbactam, tazobactam, and vaborbactam) with the corresponding antibiotics used commercially in hospitals was also tested together as the substrates of the 14 proteins. The transformant containing the pET26 vector displayed no or low‐level resistance to all antibiotics and the antibiotic and inhibitor agent combinations, similar to the observation in the transformant containing pET‐D1. In contrast, other transformants with the genes of the predicted *β*‐lactamases showed MIC values to various antibiotics, suggesting that only the D2 protein among the 10 screened enzymes was not a *β*‐lactamase. Regarding the pET‐A1 and pET‐A2 transformants in the Class A *β*‐lactamases, both showed resistance to a similar spectrum of antibiotics. The highest MIC value for the A1 and A2 transformants was to ampicillin (256 µg mL^−1^); the MIC of the characterized *β*‐lactamase (transformant with pET‐AC) to ampicillin was 512 µg mL^−1^. pET‐A1 and pET‐A2 transformants had lower MIC values to other antibiotics than the pET‐AC transformant did. In addition, the lower MIC values than those of the predicted *β*‐lactamases and characterized enzymes were detected in the Class B and D *β*‐lactamases. In the Class C *β*‐lactamases, the transformants with C1, C2, and C3 showed higher MIC values to carbenicillin, azlocillin, and aztreonam. Moreover, all combinations of antibiotics and inhibitor agents inhibited the growth of the transformants with the *β*‐lactamases discovered in this study (MIC<2 µg mL^−1^).

To further confirm the resistance of the screened *β*‐lactamases, the bacteria encoding four *β*‐lactamases, including *P. stutzeri* encoding A1 and A2, *Clostridium sporogenes* encoding B3, *Nocardiopsis dassonvillei* encoding C3, and *Schaalia odontolytica* harboring D1, were further selected for the MIC test (Figure 3c). *N. dassonvillei* is reportedly related to pulmonary infections,^[^ ^26^ ^]^ similar to *S. odontolytica*, which is typically in the oral cavity.^[^ ^27^ ^]^ The MICs of the *β*‐lactams for the four bacteria showed that they were resistant to a similar spectrum of *β*‐lactams as the transformants with the corresponding genes. However, the four strains showed increased susceptibility compared with that of the transformants. This may have been caused by the transformants with the T7 promoter being able to express abundant proteins.^[^ ^28^ ^]^

Subsequently, we selected four enzymes in each class (A1, B1, C1, and D1) to purify from *E. coli* and confirm the activity of six *β*‐lactams (Figure 3d; Figure S9, Supporting Information). A1 showed a similar catalytic efficiency to penicillin, ampicillin, carbenicillin, and cephalosporin; however, the enzyme could not hydrolyze meropenem and aztreonam. The substrate range of A1 was similar to that of D1. B1 hydrolyzed the six *β*‐lactams; however, the efficiency to aztreonam was significantly lower. Similarly, C1 did not catalyze aztreonam but hydrolyzed the other five lactams. The overall catalytic efficiency (*k*_cat_/*K*_m_) of the four enzymes was in the same range of 0.17–3.46 s^−1^ µM^−1^. In retrospect, the enzyme kinetic parameters were consistent with the resistance spectrum shown by the MIC test. These results demonstrate that nine enzymes from the ten candidates showed *β*‐lactamase activity against many antibiotics.

### 2.3 Abundance of the *β*‐*Lactamase* Genes in the Human Gut Microbiome is Correlated with Antibiotic Consumption

The emergence and spread of antibiotic‐resistant bacteria is a well‐known consequence of widespread antibiotic use.^[^ ^29^ ^]^ Additionally, the association between *β*‐lactam use and resistance in Europe has been described.^[^ ^30^ ^]^ However, the relationship between *β*‐lactam consumption and the abundance of *β‐lactamases* remains unclear globally. In this study, we aimed to investigate this association from a global perspective. We retrieved the DNA sequences of *β*‐lactamases from the clusters validated in this study and characterized from the previous studies. In total, 16202 DNA sequences were considered, excluding those in Cluster D2 (Figure 2E). The abundance of these genes was quantified through the mapping of the DNA sequences to the gut metagenomic datasets, which included 2394 healthy individuals across 19 countries (Table S2, Supporting Information).

**Figure 4:**
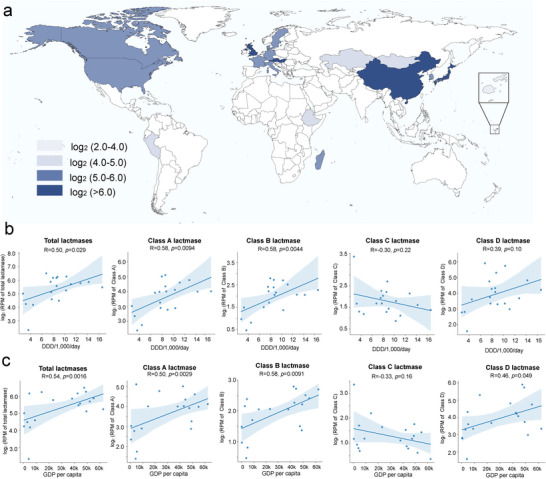
Correlation between the abundance of the microbial *β‐lactamases* in the healthy human gut and total antibiotic consumption. a) The abundance of the *β*‐lactamase genes in the gut was quantified by mapping the genes identified from those characterized in this study and previous studies to the metagenomic datasets. These datasets included 2394 healthy participants from 19 countries. The density in the map shows the average *β‐lactamase* abundance in the individuals. b) The correlation between the abundance of the gut *β‐lactamase* genes and the total *β*‐lactam consumption rate of the countries calculated using the Pearson correlation coefficient. The total antibiotic consumption rates were estimated from the defined daily doses (DDD) per 1000 population per day between 2000 and 2018. c) The correlation between the abundance of the gut *β‐lactamases* and income (gross domestic product per capita) of 19 countries between 2000 and 2018.

The data showed that the abundance of the *β*‐lactamase genes from different countries could be divided into four groups (log_2_[RPM] > 6.00, 5.00 < log_2_[RPM] < 6.00, 4.00 < log_2_[RPM] < 5.00, and log_2_[RPM] < 4.00) (**Figure** **4**a; Figure S10, Supporting Information). Individuals in Japan had the highest abundance of genes encoding *β*‐lactamase in the gut microbiome (n = 32, log_2_[RPM] = 6.52, 95% Confidence Interval [CI]: 5.78–7.45), followed by those in three European countries with log_2_(RPM)>6.00, including Austria (n = 63, log_2_[RPM] = 6.28, CI: 5.33–7.24), Hungary (n = 15, log_2_[RPM] = 6.26, CI: 5.43–7.09), and the UK (n = 211, log_2_[RPM] = 6.18, CI: 5.45–6.93). The gene abundance in the Chinese population was also at this level (n = 494, log_2_[RPM] = 6.18, CI: 6.09–6.27). A mid‐level *β‐lactamase* abundance (5.00<log_2_[RPM]<6.00) was estimated in Sweden (n = 43), Italy (n = 64), Korea (n = 24), the USA (n = 296), Germany (n = 60), France (n = 20), Denmark (n = 95), Canada (n = 90), and Madagascar (n = 170). In contrast, individuals with a relatively low abundance of *β‐lactamases* (log_2_[RPM]<5.00) were from developing countries, including Kazakhstan (n = 307, log_2_[RPM] = 4.59, CI: 4.47–4.72), Mongolia (n = 63, log_2_[RPM] = 4.48, CI: 4.16–4.81), Ethiopia (n = 50, log_2_[RPM] = 4.24, CI: 3.85–4.62), and Peru (n = 72, log_2_[RPM] = 4.18, CI: 3.92–4.43). Among the countries analyzed in this study, individuals from Fiji exhibited the lowest abundance of *β‐lactamases* (n = 225, log_2_[RPM] = 2.31, CI: 0.11–4.51). Although the total gene abundances in the populations of different countries differed, the genes belonging to Classes A and B relatively accounted for the majority of the abundance (Figure S10, Supporting Information). This data is consistent with that of a previous study, which showed that Class A and B3 *β‐lactamases* are highly abundant in the human, pig, and bovine digestive system resistomes.^[^ ^31^ ^]^ In retrospect, these data indicated that the individuals from developed countries had a high‐ or middle‐level *β‐lactamase* abundance in the gut microbiome. In contrast, the gene abundance in the gut microbiome of individuals from developing countries, except Madagascar, was relatively low. Madagascar is among the least developed countries where pneumonic plague has been endemic for several years,^[^ ^32^ ^]^ and *β*‐lactams have been used to treat the disease in the country,^[^ ^33^ ^]^ which has possibly resulted in a higher *β‐lactamase* abundance in the gut microbiome.

The different abundance levels of *β‐lactamases* across the abovementioned countries prompted us to investigate the factors affecting the abundance. The consumption of *β*‐lactams, which are the substrates of *β*‐lactamases, may influence the gene abundance in the gut microbiomes. We collected the *β*‐lactam consumption data (defined daily doses [DDD] per 1000 population per day) of 19 countries between 2000 and 2018 (Dataset S6, Supporting Information).^[^ ^34^ ^]^ The Pearson correlation coefficient analysis showed a positive correlation between *β*‐lactam consumption and the total abundance of *β‐lactamases* in the gut microbiome (R = 0.50, *p* = 0.029) (Figure 4b). In addition, there was a strong correlation between *β*‐lactam use and the abundance of Class A and B *β‐lactamases* (R = 0.58, *p* = 0.0094; R = 0.58, *p* = 0.044, respectively), while no significant correlation existed for those of Class C (R = ‐0.26, *p* = 0.28) and D (R = 0.39, *p* = 0.10) *β‐lactamases*. Our findings indicate a correlation between the abundance of *β‐lactamases* in the human gut microbiome and antibiotic consumption; however, they do not conclusively establish a causal relationship.

The difference in *β‐lactamase* abundance between developing and developed countries further prompted us to investigate whether economic factors can affect gene abundance. We collected the gross domestic product per capita data from 19 countries between 2000 and 2018 (Dataset S7, Supporting Information) and discovered a statistically significant positive relationship between income and *β*‐lactam consumption (R = 0.51, *p* = 0.024, Figure S11, Supporting Information) The correlation between antibiotic consumption and *β‐lactamase* abundance was similar to that observed between the income of different countries and gene abundance (Figure 4c). A positive correlation existed between income and total *β‐lactamases* of Classes A, B, and D (R = 0.54, *p* = 0.0016; R = 0.50, *p* = 0.0029; R = 0.58, *p* = 0.0091; R = 0.46, *p* = 0.049, respectively), while no significant correlation was observed between income and Class C *β‐lactamases* (R = ‐0.33, *p* = 0.16). These insights can guide public health strategies and interventions to reduce antibiotic consumption.

### 2.4 Alteration in the Abundance of *β‐Lactamases* Within the Gut Microbiome of Individuals with Various Diseases

**Figure 5:**
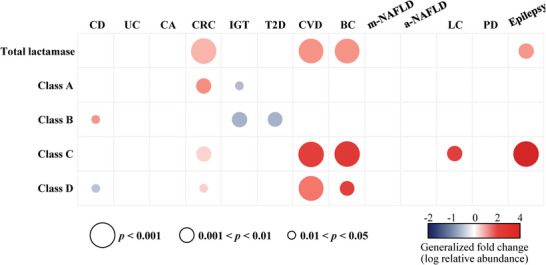
Association between the abundance of gut *β‐lactamases* and human diseases. The statistical alteration of the *β‐lactamases* between the metagenomes of healthy controls and patients was determined using the Wilcoxon rank‐sum test. Three levels of statistical significance (0.01<*p*< 0.05, 0.001<*p*<0.01, and *p*<0.001) were displayed by the size of the bubble size. A heatmap was used to display the generalized fold change in a red color to indicate an increased abundance and a blue color to indicate a decreased abundance. The definitions of the disease abbreviations are as follows: CD: Crohn's disease, UC: ulcerative colitis, CA: colorectal adenomas, CRC: colorectal cancer, IGT: impaired glucose tolerance, T2D: Type 2 diabetes, CVD: atherosclerotic cardiovascular disease, BC: Breast cancer, m‐NAFLD: mild nonalcoholic fatty liver disease, a‐NAFLD: advanced NAFLD, LC: liver cirrhosis, and PD: Parkinson's disease.

The correlation observed between the abundance of *β‐lactamases* and antibiotic consumption suggests a relationship between the abundance of *β‐lactamases* and certain disease states. To confirm this, we performed a comparative study of *β‐lactamase* abundance in 17 publicly accessible metagenomic case‐controlled cohorts to investigate possible correlations between their abundance and various diseases (Table S3, Supporting Information). These datasets covered 13 disease types, including gastrointestinal disorders (Crohn's disease (CD), Ulcerative colitis (UC), colorectal adenomas (CA), colorectal cancer (CRC)), cardiovascular diseases (CVD), metabolic conditions (impaired glucose tolerance [IGT] and Type 2 diabetes [T2D]), breast cancer (BR), liver diseases (mild nonalcoholic fatty liver disease [NAFLD], advanced NAFLD, and liver cirrhosis [LC]), and neurological disorders (Parkinson's disease [PD] and epilepsy). Our analysis comprised 1484 patient and 1412 healthy control samples. We assessed the abundance of *β‐lactamases* by aligning them with gut metagenomic datasets and determined significant differences in the abundance between the control and disease groups using the Wilcoxon rank‐sum test (**Figure** **5**; Figure S12, Supporting Information).

The oral consumption of antibiotics can cause dysbiosis, which prompted us to investigate the abundance of *β‐lactamases* in intestinal diseases, particularly CD, UC, CA, and CRC (Figure 5; Figure S12a‐d, Supporting Information). In the gut microbiomes of patients with CD (n = 239, n = 121 CTRLs) in the cohorts from USA and Netherlands, Class B *β‐lactamase* abundance was increased (*p* = 0.027, FC = 0.58) while Class B and D *β‐lactamase* abundances were decreased (*p* = 0.017, FC = ‐0.49). however, the patients with UC (n = 160, n = 121 CTRLs), no significant changes in the overall *β‐lactamase* abundance or any specific class were observed (*p*>0.05). Similarly, no significant alterations were detected in the gut microbiomes of patients with CA (n = 117) compared with those of the controls (n = 157) in the cohorts from Austria, France, and Germany (*p*>0.05). However, analyses of seven independent cohorts from Austria, China, Germany, Italy, and the USA (n = 345, n = 351 CTRLs) revealed a significant increase in the *β‐lactamase* abundance of Classes A, C, and D in the gut microbiomes of patients with CRC (*p* = 0.0011, *p* = 0.0041, and *p* = 0.019, respectively; fold‐change [FC] = 1.49, FC = 2.07, and FC = 0.35, respectively), while the abundance of the total *β‐lactamases* showed a significant increase also (*p* = 0.00016, FC = 0.82). The increased *β*‐lactamases in the gut of the patients may be caused by prophylactic antibiotic therapy.^[^ ^35^ ^]^ Additionally, dysbiosis caused by oral antibiotic use was associated with an increasing risk of CRC,^[^ ^36^ ^]^ which may also alter the composition of microbial *β‐lactamases* in the gut.

Antibiotic use could also affect glucose metabolism by altering gut microbiota.^[^ ^37^ ^]^ Thus, we compared the *β‐lactamase* abundance in the gut between the controls and patients with IGT using the dataset from Sweden (n = 49 and n = 43 CTRLs) (Figure 5; Figure S12e, Supporting Information). The results showed that Class A and B *β‐lactamase* abundances in the gut of patients with IGT were decreased (*p* = 0.010, FC = ‐0.64 and *p* = 0.010, FC = ‐0.69). Furthermore, analysis of the two T2D cohorts from Sweden and China (n = 240 and n = 226 CTRLs) showed that the abundance of Class B *β‐lactamases* in the gut of patients were decreased (*p* = 0.0076, FC = 0.26) (Figure 5; Figure S12f, Supporting Information). However, the cause for the decrease in these *β‐lactamases* was unelucidated. Some studies have shown that the prevalence of diabetes showed an inverse association with the consumption of the broad‐spectrum antibiotic penicillin,^[^ ^38a,b^ ^]^ suggesting a complex relationship among antibiotic consumption, glucose metabolism, and *β‐lactamase* abundance.

Considering that prolonged changes in the gut microbiota owing to antibiotic exposure might be associated with a higher risk of CVD,^[^ ^39^ ^]^ we further investigated the abundance of *β‐lactamases* in the gut microbiomes of patients with CVD ((Figure 5; Figure S12g, Supporting Information) using a Chinese cohort (n = 214, n = 171 CTRLs). The total abundance of *β‐lactamases* in individuals with ailments was significantly increased compared with those of the control (*p* = 5.0e‐06, FC = 0.56). This increase was primarily caused by the increased Class C and D *β‐lactamases*, which were positively associated with the disease (*p* = 4.2e‐09, FC = 1.23 and *p* = 2.5e‐05, FC = 0.72, respectively). We proposed that the increased microbial *β‐lactamases* in the gut of patients with CVD may be caused by the antibiotic consumption because antibiotic treatment has been used for the secondary prevention of atherosclerosis.^[^ ^40^ ^]^ We found that the alteration trend of gut *β‐lactamases* in participants with CVD was similar to that observed in patients with BR, where analyzing the Chinese BR cohort (n = 62, n = 71 CTRLs) showed that the total abundance of the genes was increased significantly (*p* = 9.1‐e04, FC = 0.53) with the increase from the Class C and D *β‐lactamases* (*p* = 7.7‐e08, FC = 1.23 and *p* = 1.3‐e03, FC = 0.72, respectively) (Figure 5; Figure S12h, Supporting Information). As the CVD and BR datasets were collected from the same Chinese region (South China), the same alteration in gut *β‐lactamases* may be caused by regional or subsistence‐specific variations.^[^ ^41^ ^]^ Additionally, the increased microbial *β‐lactamases* may be caused by the antibiotic consumption because *β*‐Lactam treatment has been used for the secondary prevention of atherosclerosis^[^ ^40^ ^]^ and for the prevention of infection in BR.^[^ ^42^ ^]^

Several lactam antibiotics, such as penicillin, amoxicillin/clavulanate, and cephalosporins, could cause hepatotoxicity,^[^ ^43^ ^]^ implying that the gut microbial *β‐*lactamase levels are essential for patients with liver diseases. We retrieved the metagenomic datasets with NAFLD from the USA (n = 74 mild NAFLD, n = 12 advanced NAFLD, n = 308 CTRLs) and the LC datasets from China (n = 123, n = 114 CTRLs) (Figure 5; Figure S12i‐k, Supporting Information). After examining the two cohorts (Figure 5), only the Class C *β‐lactamases* showed a notably decreased presence in the gut microbiomes of patients with LC (*p* = 0.040, FC = 1.29). The data implies that the antibiotic resistance patterns were altered in these individuals, which might influence the antibiotic choice in treating infections in patients with LC.

In addition to inhibiting infections against bacteria, lactams have been used as agents to combat neurological diseases by restoring glial glutamate transporter expression and activating glutamate—the primary excitatory neurotransmitter.^[^ ^44^ ^]^ We successfully assessed the prevalence of *β‐lactamases* in the gut microbiomes of individuals with Parkinson's disease and epilepsy (Figure 5; Figure S12l‐m, Supporting Information). For the Parkinson's disease cohort from Germany (n = 28, n = 31 CTRLs), the abundance of total *β‐lactamases* and the genes in each class showed no significant alterations (*p*>0.05). However, in the cohort of Swedish patients with epilepsy (n = 24, n = 22 CTRLs), the total abundance of microbial *β‐lactamases* was increased significantly (*p* = 0.0014, FC = 0.53), which was primarily from the Class C *β‐lactamases* (*p* = 1.0e‐06, FC = 3.67). The alteration in the abundance of *β‐lactamases* occurred in patients with epilepsy, and the data can be used to guide the usage of antibiotics for the disease.

## 3 Discussion

The widespread and mostly inappropriate use of antibiotics in human medicine, agriculture, and animal husbandry has caused an increased selection pressure on bacteria to develop and spread antibiotic resistance mechanisms, including *β*‐lactamases. The emergence and spread of *β*‐lactamase‐producing bacteria contribute to antibiotic resistance, which is a significant global health concern. Hence, the discovery and mining of novel *β*‐lactamases have generally gained attention from the scientific and medical fields. Some *β*‐lactamases, such as the New Delhi metallo‐*β*‐lactamase from *Enterobacteriaceae*,^[^ ^45^ ^]^ KPC type *β*‐Lactamases,^[^ ^46^ ^]^ AmpC *β*‐lactamases,^[^ ^47^ ^]^ and CTX‐M *β*‐lactamases,^[^ ^48^ ^]^ have caused significant global health concern. However, a panoramic view of *β*‐lactamases in the human microbiome, including the diversity, abundance, and biochemical knowledge, remains lacking. In this study, we identified and characterized nine *β*‐lactamases from bacteria colonized in humans by combining network analyses. By combining large‐scale metagenomic data from geographically diverse human cohorts, our study shows that the abundance of *β‐lactamases* in the human gut microbiome is positively associated with *β*‐lactam consumption. We observed that *β‐lactamase* abundance in the human gut is highly increased in patients with certain diseases, such as CRC and CVD. This study provides rich and novel information for tracking *β*‐lactamase diversity, understanding *β*‐lactam resistance, and guiding antibiotic stewardship.

The gene sequence named *β‐lactamase* increases exponentially in the public databases^[^ ^9^ ^]^; however, misannotation and over‐annotation of the molecular functions and the inadequate functional validation owing to computational annotation by sequence‐based similarity raises concerns on whether all of the named *β*‐lactamase genes have resistant functions. Although microbial *β*‐lactamases have a long history of experimental discovery, the verified *β*‐lactamases account for only a small fraction of all the *β*‐lactamases in nature. The functional verification of *β*‐lactamases was performed by examining individual pathogenic organisms in pure culture, while the functional metagenomics verified via screening a metagenomic library is reportedly an effective and confident method to ascribe a resistance function to known *β*‐lactamases and discover *β*‐lactamases with novel sequences; 13 *β*‐lactamases were identified from the soil using this approach.^[^ ^20^ ^]^ Using a similar approach, 32 *β*‐lactamase candidates with four novel *β*‐lactamases were identified from wildlife and their habitats.^[^ ^49^ ^]^ However, this screening method is laborious and time intensive owing to the abundant colonies screened and the low rate at which clones of interest are obtained (which rarely exceeds 6% based on a previous report).^[^ ^50^ ^]^ Moreover, the computational method was used to identify metallo‐*β*‐lactamases, and 76 novel Class B *β*‐lactamases were predicted, while 18 of the 21 tested genes were confirmed to be *β*‐lactamases.^[^ ^11^ ^]^ Nevertheless, the 70% amino acid identity cut‐off used for the Basic Local Alignment Search Tool search may have caused the loss in novelty of the hit proteins. In this study, we used an SSN analysis of the 40% identity cut‐off to cluster *β*‐lactamases from the four classes because homologous proteins above the cut‐off are more likely to share a similar function.^[^ ^51^ ^]^ Clustering methods significantly reduce the number of sequences that need to be processed individually by grouping similar sequences together. This is particularly important when dealing with large‐scale datasets, as it improves computational efficiency and saves time and computing resources. Moreover, clustering helps identify and distinguish functionally similar or related protein families. By grouping proteins with similar functions into the same cluster, clustering reveals the biological significance and evolutionary relationships of protein families. This approach not only enhances the interpretability of the data but also facilitates more comprehensive analyses of protein functions and interactions.

Together with the genome context and the signal peptide characteristics of *β*‐lactamase, we discovered several novel clusters and sequences within Class A (one cluster: 79 sequences), B (three clusters: 80, 41, and 15 sequences, respectively), C (three clusters: 162, 104, and 52 sequences, respectively), and D (two clusters: 630 and 154 sequences, respectively) *β*‐lactamases. Subsequent to experimental validation, it was confirmed that the protein from the second cluster of Class D exhibited no lactam activity, while nine of the ten candidate enzymes demonstrated *β*‐lactamase activity. The notably high success rate can be attributed to several factors: 1) the use of databases with precise and detailed annotations, including DOI numbers; 2) the adoption of a multi‐tiered selection methodology, encompassing techniques such as SSN, GNN, and signal peptide analysis, to effectively sift through potential sequences. A similar high success rate was also reported in mining haloalkane dehalogenases from the database, which yielded 40 active enzymes from 45 genes.^[^ ^52^ ^]^ We identified 1163 unique *β*‐lactamases belonging to eight clusters (Figure S13, Supporting Information), suggesting that our approach is efficient and reliable. As research on antibiotic resistance and metagenomics has evolved, we propose that this approach enables the mining of other resistant genes and is crucial for enhancing the global surveillance of antibiotic resistance.

Our novel proteins were primarily from Proteobacteria (33%), Bacteroidetes (22%), Firmicutes (17%), Actinobacteria (8%), and Verrucomicrobia (5%) (Figure S13a, Supporting Information). The ratio of the sources of the newly identified *β*‐lactamases differed from that of the previously characterized enzymes, which were primarily from Proteobacteria (89%). The comparison suggests that our study expanded the spectrum of *β*‐lactamase producing bacteria at the phylum level. The abundance from the HMP dataset of the healthy human gut microbiome showed that the newly identified *β*‐lactamases in Class D ranked first, followed by those in Classes C, B, and A (Figure S13b, Supporting Information), which also differed from the previously characterized enzymes, where the Class A *β*‐lactamases showed the highest abundance in the same dataset (Figure 2c). These differences suggest that the human gut microbiome is a rich reservoir with a high level of unknown *β*‐lactamases.

Many clinically relevant *β*‐lactamases have evolutionary origins in environmental microorganisms and spread through mobile genetic elements, such as plasmids, integrative conjugative elements, and transposons.^[^ ^9^ ^]^ We surveyed the genetic context upstream and downstream of the novel *β*‐lactamases (Figure S14, Supporting Information) and found no mobility element syntenic nearby, foreshadowing that the newly identified *β*‐lactamases may only be future threats for *β*‐lactam resistance. We observed that *β*‐lactamases, predominantly chromosomal, lack the ability for horizontal gene transfer, indicating that differences in their abundance are associated with changes in the host's relative abundance rather than spread to additional hosts. This is further supported by the fact that shifts in microbial community structure can significantly influence the abundance of these genes. For example, the impact of microbial community changes on the abundance of antibiotic‐resistance genes, including *β‐lactamases*.^[^ ^53^ ^]^ Moreover, Calderón‐Franco et al. emphasize the influence of microbial community dynamics on *β‐lactamase* distribution.^[^ ^54^ ^]^ These findings align with the assertion that changes in *β‐lactamase* gene abundance are likely secondary to broader ecological shifts in bacterial populations rather than a direct result of antibiotic consumption. Despite this, some of the *β*‐lactamases in the new clusters were from pathogens that may have global health impacts, such as *Porphyromonas gingivalis*, which encodes a Class C *β*‐lactamases identified in this study (A0A0K2J4×4). *P. gingivalis* is a major contributor to periodontitis (gum disease), which is a common oral health problem affecting a large portion of the global population.^[^ ^55^ ^]^ When these enzymes are produced in large quantities, they can play a role in developing resistance to carbapenems, particularly when this overproduction is coupled with reduced permeability of the outer membrane and/or heightened activity of efflux pumps. Additionally, the *Rickettsiaceae* bacterium, harboring a Class D *β*‐lactamase (A0A1W9SC55), causes Rocky Mountain spotted fever and typhus globally.^[^ ^56^ ^]^ Thus, our study may pre‐estimate a future risk of *β*‐lactam resistance.

After the investigations that confirmed our method could be used to discover diverse, unreported *β*‐lactamases, we used the characterized clusters in our study and previously characterized clusters, which can reduce false alignments, to analyze *β‐lactamase* abundance in the human gut. Briefly, in the healthy participants, the population from developed countries showed a relatively higher abundance of *β‐lactamases* than the population from developing countries. Furthermore, the abundance was positively related to *β*‐lactam consumption and income. A recent study based on ten countries showed highly significant correlations between the total antibiotic resistance gene (ARG) abundance and antibiotic usage rates and that only *β*‐lactam consumption was correlated with the ARGs related to *β*‐lactams.^[^ ^57^ ^]^ The ten countries selected in the study were developed countries, except Kazakhstan, and the antibiotic consumption rates in the study were from the Center for Disease Dynamics, Economics & Policy and the World Health Organization (WHO) reports. However, the WHO report was not available for many countries, which limited the analysis. In our study, we also considered several low‐income and middle‐income countries and used modeling to analyze antibiotic consumption data, which incorporates antibiotic usage and consumption data.^[^ ^34^ ^]^ Hence, our data further demonstrated a correlation with the details and showed that the abundance of Class A and B *β‐lactamases* contributed to the significant correlation and that the abundance was related to income. While Class C *β‐lactamases* behave differently than Class A and B *β‐lactamases*, this difference can be attributed to their typical genomic locations. Class C *β‐lactamases* are usually encoded on the chromosomes of many Enterobacteriaceae and some other bacteria, whereas Class A and B *β‐lactamases* are commonly found on plasmids. Thus, according to the research, we proposed that the availability and accessibility of *β*‐lactams in developed countries are higher than that in developing countries,^[^ ^58^ ^]^ which causes increasing selection pressure for resistant *β*‐lactamase‐encoding bacteria within the gut microbiota of the populations. Notably, the infections caused by these resistant bacteria may be more challenging to treat, leading to higher healthcare costs and prompting the need for caution.

After proposing the association between the abundance of *β*‐lactamases in the gut and the availability and accessibility of *β*‐lactams at both national and population levels, we aimed to investigate whether this relationship also exists on a smaller scale and at the individual level. We utilized two datasets from curatedMetagenomicData (Table S4, Supporting Information),^[^ ^59^ ^]^ which provided information on antibiotic uptake. In the datasets from Denmark, healthy individuals who had taken meropenem, vancomycin, or gentamicin (n = 12) were compared to a control group (n = 12) who had not taken any antibiotics (Figure S15a, Supporting Information). Although those who had taken antibiotics exhibited relatively higher levels of total *β*‐lactamases, statistical analysis revealed no significant difference between the two groups. In the dataset from Spain and Germany, patients with pancreatitis who had taken antibiotics (specific antibiotics unknown) (n = 19, n = 11 CTRL) were studied. The data showed a similar trend where patients on antibiotics had relatively higher *β*‐lactamase abundance, but statistical analysis again indicated no significant difference (Figure S15b, Supporting Information). The differing results between national and smaller‐scale levels may be due to variations in antibiotic usage among individuals; some may use antibiotics for a short period, while others may use them for longer durations. These differences can affect the accumulation and abundance of resistant strains in the gut microbiota. Additionally, studies at the individual level typically have smaller sample sizes, which may result in an inability to detect subtle but real differences.

We further applied the characterized *β*‐lactamase sequences to analyze the gene abundance in the gut microbiome of patients with ailments. The abundance of total *β‐lactamases* or *β‐lactamases* from some classes was increased substantially in the gut microbiome of patients with CRC, CVD, and BC. This increase can be attributed to the frequent antibiotic treatments these patients receive, which select for antibiotic‐resistant bacteria harboring *β*‐lactamase genes, similar to the trend seen in populations of developed countries. The increased *β‐lactamases* in the patients may also be associated with the alteration in the gut microbiome during disease, as gut microbiota dysbiosis in diseases has been studied and recognized.^[^ ^36^, ^39^ ^]^ The gut microbiome is a complex ecosystem with many interacting microorganisms. Certain diseases may alter the structure of the gut microbiome, leading to an increase in the abundance of *β*‐lactamase genes. However, this change may be indirect and due to the reorganization of the microbial ecosystem caused by the disease, rather than being directly caused by the disease itself. However, the increased *β‐lactamases* in patients related to any factors may affect treatment, whether it is a causal relationship caused by antibiotic use or an association with dysbiosis. Such changes complicate the management of secondary infections and negatively affect patient responses to cancer treatments, such as chemotherapy or immunotherapy.

This study has certain limitations. First, we may have missed a few experimentally characterized *β*‐lactamases from previous studies, as *β*‐lactamases are among the most studied enzymes. Second, the structure of some newly identified *β*‐lactamases should be elucidated to investigate the detailed catalytic mechanisms. Third, when we analyzed the abundance of *β‐lactamases* with diseases, some datasets were only from a single region; therefore, whether the relationship setup in this study is region‐specific or can be shared across other regions requires further investigation. Fourth, physiological experiments should be performed to study the function of the *β*‐lactamases in the gut of model animals.

## 4 Conclusion

Our study highlights the effectiveness of integrating network analysis techniques with experimental validation in identifying *β*‐lactamases. Our proof‐of‐concept research demonstrates a reliable approach for discovering new enzymes, providing a more efficient alternative to traditional experiment‐based methods. Coupled with the discovery of new sequences, our study reveals a notable association between the abundance of *β*‐lactamases and antibiotic consumption, as indicated by the analyzed sequences, without asserting a definitive causal relationship. Noting that an increased abundance of *β‐lactamases* was also observed in patients with certain diseases is essential. These findings contribute valuable insights into the antibiotic resistance study, antibiotic stewardship, and clinical practice.

## 5 Experimental Section

### *β*‐*Lactamase* Sequence Mining and in Silico Analysis

The experimentally characterized microbial *β*‐lactamases were meticulously collected from the Swiss‐Prot database (status: reviewed) and the BLDB. During the integration of *β*‐lactamase data from these two sources, each sequence's origin was cross‐referenced to ensure completeness. Redundant sequences were eliminated using CD‐HIT, and the remaining sequences were further validated with PubMed identifiers.^[^ ^60^ ^]^ For the construction of the HMM profiles, a multiple sequence alignment of the *β*‐lactamase sequences from each class was first performed using Muscle. Utilizing these aligned sequences, specific HMM profiles for each class were then created employing HMMER 3.3.1, which utilized known sequences, effectively “trained” the HMM profiles to capture the essential features of each *β*‐lactamase class.^[^ ^61^ ^]^ The HMM profiles were subsequently employed as queries for the HMMER search (e‐value = 1e‐15) against the UniProt and InterPro databases, with a cut‐off date of March 2020. The obtained *β*‐lactamase homologs are listed in Dataset S1 (Supporting Information). A protein sequence similarity network (SSN) was build employing the Enzyme Function Initiative‐Enzyme Similarity Tool (EFI‐EST)^[^ ^62^ ^]^ and visualized it with Cytoscape 3.8.^[^ ^63^ ^]^ In the network, each node represents a protein, and an edge connecting two proteins signifies an e‐value below the chosen threshold. SignalP 6.0 was utilized to predict the signal peptides of the sequences.^[^ ^64^ ^]^

### Analysis of the Abundance of *β*‐*Lactamases* in Human Gut Metagenomes

Short, Better Representative Extract Dataset (ShortBRED) was employed in the built‐in EFI‐chemically guided functional profiling (EFI‐CGFP) platform to create representative protein markers, identify and quantify *β*‐lactamases in human metagenomes from the Human Microbiome Project (HMP) as previously reported.^[^ ^65^ ^]^ To measure the abundance from other datasets, DNA sequences of *β‐lactamases* were retrieved from the clusters that were validated in this study and characterized from previous studies; for example, the sequences in Cluster 4 of Class D were not included as the proteins were not *β*‐lactamases. The metagenomic data were downloaded from the GenBank Sequence Read Archive and listed in Table S2 (Supporting Information) for the healthy subjects and Table S3 (Supporting Information) for individual disease samples with corresponding healthy controls. Only data without antibiotic/probiotic treatment in 3 months were considered. Sequencing reads were processed with Sickle, applying a quality cut‐off of 30 and a minimum length of 50 bp (https://github.com/najoshi/sickle). High‐quality reads were aligned to *β*‐lactamase sequences using the default settings of the Burrows‐Wheeler Alignment (BWA) MEM algorithm.^[^ ^66^ ^]^ The reads were further filtered by the SAMtools program and only reads above a mapping quality score of 1 were retained.^[^ ^67^ ^]^ Reads corresponding to each *β*‐lactamase class were counted using BEDtools (https://bedtools.readthedocs.io/). To normalize the read counts, counts reads per million (RPM) were calculated using the ggplot2 package (v3.1.0) in R. For the data from multiple cohorts, MaAsLin2 was used for mixed‐effects model analysis.^[^ ^68^ ^]^

### Expression and Functional Assay of *β*‐*Lactamases*

The DNA sequences of the selected *β‐lactamases* were tailored for *E. coli* expression by Optimizer^[^ ^69^ ^]^ and these genes were then integrated into the pET26b plasmid and introduced into BL21 (DE3) Star cells.^[^ ^70^ ^]^ *E. coli* cells containing the transformants were grown in 50 mL of LB medium with kanamycin (50 µg mL^−1^) at 37 °C for 3 h. Once the OD600 reached 0.7, protein expression was induced by adding 1 mM isopropyl‐*β*‐D‐thiogalactopyranoside (IPTG). Following 16 h of agitation at 16 °C during cultivation, the cells were collected via centrifugation at 4 °C for 10 min, and protein expression was confirmed through SDS‐PAGE analysis.

For protein purification, the cell pellets were reconstituted in a highly concentrated solution consisting of 30 mM Tris and 20% w/v sucrose (pH 8), in a 25 mL volume, and incubated for 30 min at 4 °C. Following centrifugation, the resulting supernatant was gathered, centrifuged again, and applied to a Ni‐NTA column. The column was then cleansed with lysis buffer (50 mM HEPES at pH 7.5, 300 mM NaCl, and 20 mM imidazole) before the *β*‐lactamases were extracted using 250 mM imidazole. Susceptibility was determined using the liquid broth dilution tests with Mueller‐Hinton Broth in accordance with the guidelines put forth by the Clinical and Laboratory Standards Institute (CLSI, 2017).^[^ ^71^ ^]^ The *E. coli* strains with the pET26 vector and the known *β*‐lactamases (AC, BC, CC, and DC) were employed as the positive and negative controls. To analyze antibiotic kinetics, enzyme reaction parameters were assessed by altering the antibiotic concentrations at 25 °C in the assay buffer (10 mM HEPES, 200 mM NaCl, pH 7.5). Regarding the Class B *β*‐lactamase, 0.01 mM ZnCl_2_ was supplemented in the buffer. The absorbance wavelengths for the antibiotic's degradation test were 235 nm for penicillin G, oxacillin, ampicillin, amoxicillin, carbenicillin and azlocillin; 260 nm for cephalosporin, cefaclor, ceftriaxone, and cefpodoxime; and 300 nm for meropenem and aztreonam, respectively. Enzyme kinetics were determined through the application of the Michaelis‐Menten equation using R.

### Statistical Analysis

R was used for all statistical evaluations. The Mann‐Whitney test was applied to determine the significance between two subjects. Additionally, the log‐based general fold change was computed for values between subjects using quantile intervals from 0.1 to 0.9 and increasing by 0.1 increments.^[^ ^72a,b^ ^]^

## Conflict of Interest

The authors declare no conflict of interest.

## Author Contributions

B.J., J.H.B., and J.K.L. contributed equally to this work. B.J. and C.O.J. led on design and conceptualization of the study, with input from all authors. B.J., J.H.B., J.K.L., J.Y.J., Y.S., K.H.K., J.Y.J., and C.O.J. contributed to obtaining, analyzing, and interpreting the data. B.J., J.H.B., and J.K.L. did all data analysis and laboratory work. All authors have verified all the data. B.J. and C.O.J. drafted the Article. All authors approved the final version of the manuscript had final responsibility for the decision to submit for publication.

## Supporting information



Supporting Information

## Data Availability

The data that support the findings of this study are available in the supplementary material of this article.
