# Supplementary material for: Expanding the β‐Lactamase Family in the Human Microbiome
Source: Adv Sci (Weinh). 2024 Oct 24;11(46):2403563. doi: 10.1002/advs.202403563 (PMC11633517; doi:10.1002/advs.202403563)
Supplement: Supplementary file 1 — Supporting Information [file ADVS-11-2403563-s001.pdf]

ADVANCED SCIENCE
Open Access

Supporting Information

for *Adv. Sci.*, DOI 10.1002/advs.202403563

Expanding the β-Lactamase Family in the Human Microbiome

Baolei Jia, Ju Hye Baek, Jae Kyeong Lee, Ying Sun, Kyung Hyun Kim, Ji Young Jung and Che Ok Jeon**

Supporting Information

Expanding the β-Lactamase Family in the Human Microbiome

Baolei Jia*, Ju Hye Baek, Jae Kyeong Lee, Ying Sun, Kyung Hyun Kim, Ji Young Jung, Che Ok Jeon*

B. Jia

Xianghu Laboratory, Hangzhou 311231, China

E-mail: jiabaolei@xhlab.ac.cn

B. Jia, J.H. Baek, J.K. Lee, C.O. Jeon

Department of Life Science, Chung-Ang University, Seoul 06974, Republic of Korea
E-mail: cojeon@cau.ac.kr

Y. Sun

Department of Veterinary and Animal Sciences, University of Copenhagen, Copenhagen 1870, Denmark

K.H. Kim

Department of Biological Sciences and Biotechnology, Hannam University, Daejon 34054, Republic of Korea

J.Y. Jung

Microbial Research Department, Nakdonggang National Institute of Biological Resources, Gyeongsangbuk-do 37242, Republic of Korea

Table S1. **The β-lactamases that characterized in the previous study and used for control in this study (related to figure 3).**

Uniprot ID	Class	Organism	Length	References
P28585	A	*Escherichia coli*	291	(Bauernfeind et al., 1996)
P25910	B	*Bacteroides fragilis*	249	(Rasmussen et al., 1990)
P85302	C	*Pseudomonas fluorescens*	358	(Michaux et al., 2008)
P0A1V8	D	*Salmonella typhimurium*	275	(Ledent and Frère, 1993)

References:

Bauernfeind, A., Stemplinger, I., Jungwirth, R., Ernst, S., and Casellas, J.M. (1996). Sequences of beta-lactamase genes encoding CTX-M-1 (MEN-1) and CTX-M-2 and relationship of their amino acid sequences with those of other beta-lactamases. *Antimicrob Agents Chemother* 40**,** 509-513.

Ledent, P., and Frère, J.M. (1993). Substrate-induced inactivation of the OXA2 beta-lactamase. *Biochem J* 295 (Pt 3)**,** 871-878.

Michaux, C., Massant, J., Kerff, F., Frère, J.M., Docquier, J.D., et al. (2008). Crystal structure of a cold-adapted class C beta-lactamase. *Febs j* 275**,** 1687-1697.

Rasmussen, B.A., Gluzman, Y., and Tally, F.P. (1990). Cloning and sequencing of the class B beta-lactamase gene (ccrA) from Bacteroides fragilis TAL3636. *Antimicrob Agents Chemother* 34**,** 1590-1592.

Table S2. **The metagenomic datasets used to analyze β-lactamases in the healthy participants in this study (related to figure 4).**

Accession Number	sample size	Country	Reference
PRJEB7774	63	Austria	(Feng et al., 2015)
PRJNA297252	90	Canada	(Vincent et al., 2016)
PRJEB6337	112	China	(Qin et al., 2014)
PRJNA453965	71	China	(Zhu et al., 2018)
PRJEB21528	109	China	(Jie et al., 2017)
RRJNA422434	157	China	(Qin et al., 2012)
PRJNA328899	45	China	(Liu et al., 2016)
PRJEB4336	95	Denmark	(Le Chatelier et al., 2013)
PRJNA504891	50	Ethiopia	(Pasolli et al., 2019)
PRJNA217052	225	Fiji	(Brito et al., 2016)
PRJEB6070	20	France	(Zeller et al., 2014)
PRJEB17632	60	Germany	(Costea et al., 2017)
PRJNA668745	15	Hungary	(Marfil-Sanchez et al., 2021)
PRJEB25514	40	Italy	(Wu et al., 2019)
PRJNA553191	24	Italy	(Rampelli et al., 2020)
PRJDB3601	32	Japan	(Nishijima et al., 2016)
PRJEB17632	307	Kazakhstan	(Costea et al., 2017)
PRJEB17896	24	Korea	(Kim et al., 2020)
PRJNA485056	170	Madagascar	(Pasolli et al., 2019)
PRJNA328899	63	Mongolia	(Liu et al., 2016)
PRJNA268964	72	Peru	(Obregon-Tito et al., 2015)
PRJEB1786	43	Sweden	(Karlsson et al., 2013)
PRJEB9584	211	UK	(Xie et al., 2016)
PRJNA268964	296	USA	(Obregon-Tito et al., 2015)

References:

Brito, I.L., Yilmaz, S., Huang, K., Xu, L., Jupiter, S.D., et al. (2016). Mobile genes in the human microbiome are structured from global to individual scales. *Nature* 535**,** 435-439.

Costea, P.I., Coelho, L.P., Sunagawa, S., Munch, R., Huerta-Cepas, J., et al. (2017). Subspecies in the global human gut microbiome. *Mol Syst Biol* 13**,** 960.

Feng, Q., Liang, S., Jia, H., Stadlmayr, A., Tang, L., et al. (2015). Gut microbiome development along

the colorectal adenoma-carcinoma sequence. *Nat Commun* 6, 6528.
Jie, Z., Xia, H., Zhong, S.L., Feng, Q., Li, S., et al. (2017). The gut microbiome in atherosclerotic cardiovascular disease. *Nat Commun* 8, 845.
Karlsson, F.H., Tremaroli, V., Nookaew, I., Bergstrom, G., Behre, C.J., et al. (2013). Gut metagenome in European women with normal, impaired and diabetic glucose control. *Nature* 498, 99-103.
Kim, J.Y., Whon, T.W., Lim, M.Y., Kim, Y.B., Kim, N., et al. (2020). The human gut archaeome: identification of diverse haloarchaea in Korean subjects. *Microbiome* 8, 114.
Le Chatelier, E., Nielsen, T., Qin, J., Prifti, E., Hildebrand, F., et al. (2013). Richness of human gut microbiome correlates with metabolic markers. *Nature* 500, 541-546.
Liu, W., Zhang, J., Wu, C., Cai, S., Huang, W., et al. (2016). Unique Features of Ethnic Mongolian Gut Microbiome revealed by metagenomic analysis. *Sci Rep* 6, 34826.
Marfil-Sanchez, A., Seelbinder, B., Ni, Y., Varga, J., Berta, J., et al. (2021). Gut microbiome functionality might be associated with exercise tolerance and recurrence of resected early-stage lung cancer patients. *PLoS One* 16, e0259898.
Nishijima, S., Suda, W., Oshima, K., Kim, S.W., Hirose, Y., et al. (2016). The gut microbiome of healthy Japanese and its microbial and functional uniqueness. *DNA Res* 23, 125-133.
Obregon-Tito, A.J., Tito, R.Y., Metcalf, J., Sankaranarayanan, K., Clemente, J.C., et al. (2015). Subsistence strategies in traditional societies distinguish gut microbiomes. *Nat Commun* 6, 6505.
Pasolli, E., Asnicar, F., Manara, S., Zolfo, M., Karcher, N., et al. (2019). Extensive Unexplored Human Microbiome Diversity Revealed by Over 150,000 Genomes from Metagenomes Spanning Age, Geography, and Lifestyle. *Cell* 176, 649-662 e620.
Qin, J., Li, Y., Cai, Z., Li, S., Zhu, J., et al. (2012). A metagenome-wide association study of gut microbiota in type 2 diabetes. *Nature* 490, 55-60.
Qin, N., Yang, F., Li, A., Prifti, E., Chen, Y., et al. (2014). Alterations of the human gut microbiome in liver cirrhosis. *Nature* 513, 59-64.
Rampelli, S., Soverini, M., D'amico, F., Barone, M., Tavella, T., et al. (2020). Shotgun Metagenomics of Gut Microbiota in Humans with up to Extreme Longevity and the Increasing Role of Xenobiotic Degradation. *mSystems* 5.
Vincent, C., Miller, M.A., Edens, T.J., Mehrotra, S., Dewar, K., et al. (2016). Bloom and bust: intestinal microbiota dynamics in response to hospital exposures and Clostridium difficile colonization or infection. *Microbiome* 4, 12.
Wu, L., Zeng, T., Zinellu, A., Rubino, S., Kelvin, D.J., et al. (2019). A Cross-Sectional Study of Compositional and Functional Profiles of Gut Microbiota in Sardinian Centenarians. *mSystems* 4.
Xie, H., Guo, R., Zhong, H., Feng, Q., Lan, Z., et al. (2016). Shotgun Metagenomics of 250 Adult Twins Reveals Genetic and Environmental Impacts on the Gut Microbiome. *Cell Syst* 3, 572-584 e573.
Zeller, G., Tap, J., Voigt, A.Y., Sunagawa, S., Kultima, J.R., et al. (2014). Potential of fecal microbiota for early-stage detection of colorectal cancer. *Mol Syst Biol* 10, 766.
Zhu, J., Liao, M., Yao, Z., Liang, W., Li, Q., et al. (2018). Breast cancer in postmenopausal women is associated with an altered gut metagenome. *Microbiome* 6, 136.

Table S3. **Metagenomic datasets used to analyze the abundance β-lactamases in disease samples included in this study (related to figure 5).**

**Accession Number**	**Health and Disease sample amounts**	**Country**	**Reference**
PRJNA389280	Healthy (65); CD (151); UC (84)	USA	(Schirmer et al., 2018)
PRJNA400072	Healthy (56); CD (88); UC (76)	Netherlands; USA	(Franzosa et al., 2019)
PRJEB7774	Healthy (63); CA (47); CRC (46)	Austria	(Feng et al., 2015)
PRJEB6070	Healthy (66); CA (42); CRC (91)	France; Germany	(Zeller et al., 2014)
PRJEB12449	Healthy (52); CRC (52)	USA	(Vogtmann et al., 2016)
PRJEB10878	Healthy (54); CRC (74)	China	(Yu et al., 2017)
PRJNA389927	Healthy (28); CA (28); CRC (28)	USA; Canada	(Hannigan et al., 2018)
PRJNA447983	Healthy (28); CRC (32)	Italy	(Thomas et al., 2019)
PRJEB27928	Healthy (60); CRC (22)	Germany	(Wirbel et al., 2019)
PRJEB1786	Healthy (43); IGT (49); T2D (53)	Sweden	(Karlsson et al., 2013)
PRJNA422434	Healthy (183); T2D (187)	China	(Qin et al., 2012)
PRJEB21528	Healthy (171); ACVD (214)	China	(Jie et al., 2017)
PRJNA453965	Healthy (71); BC (62)	China	(Zhu et al., 2018)
PRJNA354235 PRJNA373901	Healthy (308); mild NAFLD (74); advanced NAFLD (12)	USA	(Loomba et al., 2017;Abu-Ali et al., 2018)
PRJEB6337	Healthy (114); LC (123)	China	(Qin et al., 2014)
PRJEB17784	Healthy (28); PD (31)	Germany	(Bedarf et al., 2017)
PRJEB28847	Healthy (22); epilepsy (24)	Sweden	(Lindefeldt et al., 2019)

The sample numbers used in the analysis are shown after the cases. Abbreviations: UC: ulcerative colitis, CD: Crohn's disease, CA: colorectal adenomas, CRC: colorectal cancer, NAFLD: non-alcoholic fatty liver disease, LC: liver cirrhosis, ACVD: atherosclerotic cardiovascular disease, PD: Parkinson's disease, T2D: Type 2 diabetes, BC: Breast cancer.

References:

Abu-Ali, G.S., Mehta, R.S., Lloyd-Price, J., Mallick, H., Branck, T., et al. (2018). Metatranscriptome of human faecal microbial communities in a cohort of adult men. *Nat Microbiol* 3**,** 356-366.

Bedarf, J.R., Hildebrand, F., Coelho, L.P., Sunagawa, S., Bahram, M., et al. (2017). Functional implications of microbial and viral gut metagenome changes in early stage L-DOPA-naïve Parkinson's disease patients. *Genome Medicine* 9**,** 39.

Feng, Q., Liang, S., Jia, H., Stadlmayr, A., Tang, L., et al. (2015). Gut microbiome development along the colorectal adenoma–carcinoma sequence. *Nature Communications* 6**,** 6528.

Franzosa, E.A., Sirota-Madi, A., Avila-Pacheco, J., Fornelos, N., Haiser, H.J., et al. (2019). Gut

microbiome structure and metabolic activity in inflammatory bowel disease. *Nature microbiology*, 4, 293-305.

Hannigan, G.D., Duhaime, M.B., Ruffin, M.T., Koumpouras, C.C., and Schloss, P.D. (2018). Diagnostic Potential and Interactive Dynamics of the Colorectal Cancer Virome. *mBio* 9**,** e02248-02218.

Jie, Z., Xia, H., Zhong, S.-L., Feng, Q., Li, S., et al. (2017). The gut microbiome in atherosclerotic cardiovascular disease. *Nature Communications* 8**,** 845.

Karlsson, F.H., Tremaroli, V., Nookaew, I., Bergstrom, G., Behre, C.J., et al. (2013). Gut metagenome in European women with normal, impaired and diabetic glucose control. *Nature* 498**,** 99-103.

Le Chatelier, E., Nielsen, T., Qin, J., Prifti, E., Hildebrand, F., et al. (2013a). Richness of human gut microbiome correlates with metabolic markers. *Nature* 500**,** 541-546.

Le Chatelier, E., Nielsen, T., Qin, J., Prifti, E., Hildebrand, F., et al. (2013b). Richness of human gut microbiome correlates with metabolic markers. *Nature* 500**,** 541-546.

Lindefeldt, M., Eng, A., Darban, H., Bjerkner, A., Zetterström, C.K., et al. (2019). The ketogenic diet influences taxonomic and functional composition of the gut microbiota in children with severe epilepsy. *npj Biofilms and Microbiomes* 5**,** 5.

Loomba, R., Seguritan, V., Li, W., Long, T., Klitgord, N., et al. (2017). Gut Microbiome-Based Metagenomic Signature for Non-invasive Detection of Advanced Fibrosis in Human Nonalcoholic Fatty Liver Disease. *Cell Metab* 25**,** 1054-1062.e1055.

Qin, J., Li, Y., Cai, Z., Li, S., Zhu, J., et al. (2012). A metagenome-wide association study of gut microbiota in type 2 diabetes. *Nature* 490**,** 55.

Qin, N., Yang, F., Li, A., Prifti, E., Chen, Y., et al. (2014). Alterations of the human gut microbiome in liver cirrhosis. *Nature* 513**,** 59.

Schirmer, M., Franzosa, E.A., Lloyd-Price, J., Mciver, L.J., Schwager, R., et al. (2018). Dynamics of metatranscription in the inflammatory bowel disease gut microbiome. *Nature Microbiology* 3**,** 337-346.

Thomas, A.M., Manghi, P., Asnicar, F., Pasolli, E., Armanini, F., et al. (2019). Metagenomic analysis of colorectal cancer datasets identifies cross-cohort microbial diagnostic signatures and a link with choline degradation. *Nature Medicine* 25**,** 667-678.

Vogtmann, E., Hua, X., Zeller, G., Sunagawa, S., Voigt, A.Y., et al. (2016). Colorectal Cancer and the Human Gut Microbiome: Reproducibility with Whole-Genome Shotgun Sequencing. *PLOS ONE* 11**,** e0155362.

Wirbel, J., Pyl, P.T., Kartal, E., Zych, K., Kashani, A., et al. (2019). Meta-analysis of fecal metagenomes reveals global microbial signatures that are specific for colorectal cancer. *Nature Medicine* 25**,** 679-689.

Yu, J., Feng, Q., Wong, S.H., Zhang, D., Liang, Q.Y., et al. (2017). Metagenomic analysis of faecal microbiome as a tool towards targeted non-invasive biomarkers for colorectal cancer. *Gut* 66**,** 70-78.

Zeller, G., Tap, J., Voigt, A.Y., Sunagawa, S., Kultima, J.R., et al. (2014). Potential of fecal microbiota for early-stage detection of colorectal cancer. *Molecular Systems Biology* 10**,** 766.

Zhu, J., Liao, M., Yao, Z., Liang, W., Li, Q., et al. (2018). Breast cancer in postmenopausal women is associated with an altered gut metagenome. *Microbiome* 6**,** 136.

Table S4. **Metagenomic datasets used to analyze the abundance of β-lactamases in response to antibiotic use (related to figure S15).**

Accession Number	Health and Disease sample amounts	Antibiotic treatment (type)	Country	Reference
PRJEB38625; PRJEB42013	Healthy (61); Pancreatitis (30); PDAC (110)	Yes (Healthy; 29, Pancreatitis; 19, PDAC; 49); No (Healthy; 32, Pancreatitis; 11, PDAC; 61)	Spain; Germany	(Kartal *et al.*, 2022)
PRJEB20800	Healthy (12)	Yes (12; meropenem, vancomycin, gentamicin); No (12)	Denmark	(Palleja *et al.*, 2018)

The sample numbers used in the analysis are shown after the cases. Abbreviations: PDAC, Pancreatic ductal adenocarcinoma.

References:

Kartal, E., Schmidt, T.S., Molina-Montes, E., Rodríguez-Perales, S., Wirbel, J., et al. (2022). A faecal microbiota signature with high specificity for pancreatic cancer. *Gut*, 71, 1359-1372.

Palleja, A., Mikkelsen, K.H., Forslund, S.K., Kashani, A., Allin, K.H., et al. (2018). Recovery of gut microbiota of healthy adults following antibiotic exposure. *Nature microbiology*, 3, 1255-1265.

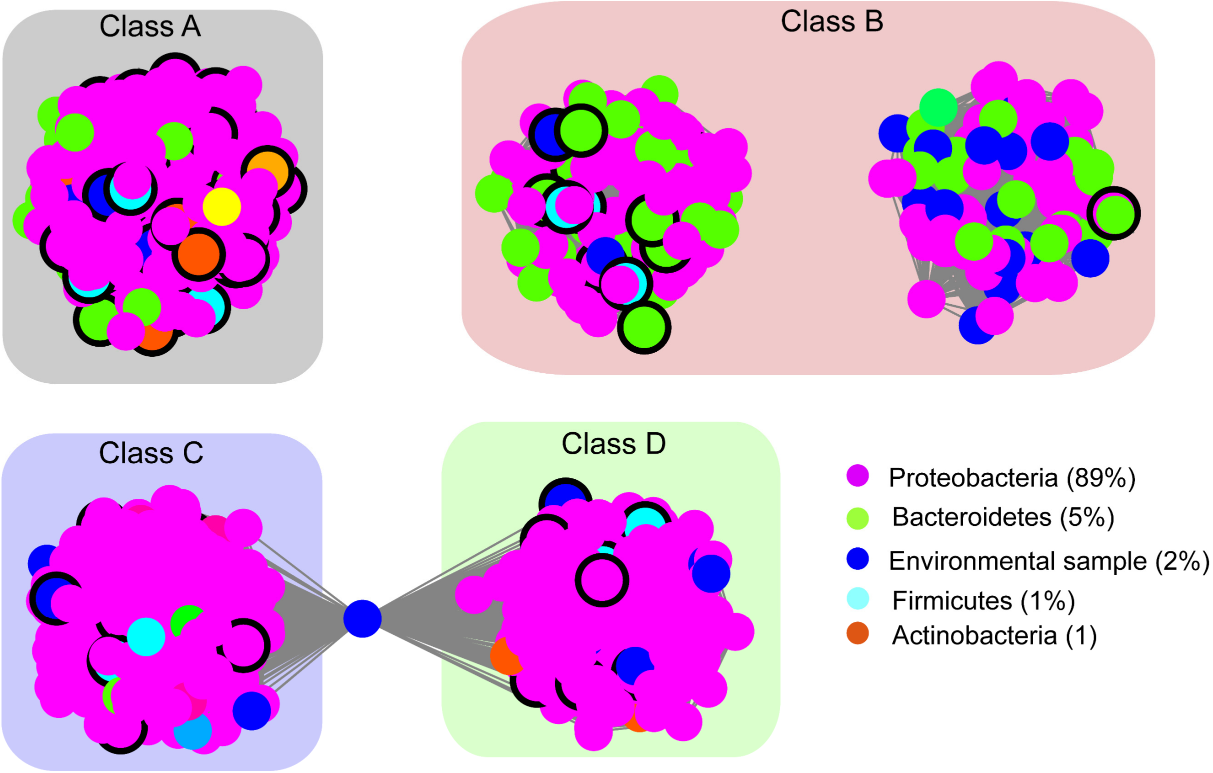

Figure S1. Sequence similarity network if 134 known β-lactamases and 1312 known β-lactamases from BLDB database using 20% cutoff of sequence identity. The proteins from SWISS-prot database were enlarged and labeled by a black circle. Proteins from the same phylum are painted by the same color and the percentage from the phylum is listed at the bottom-right.

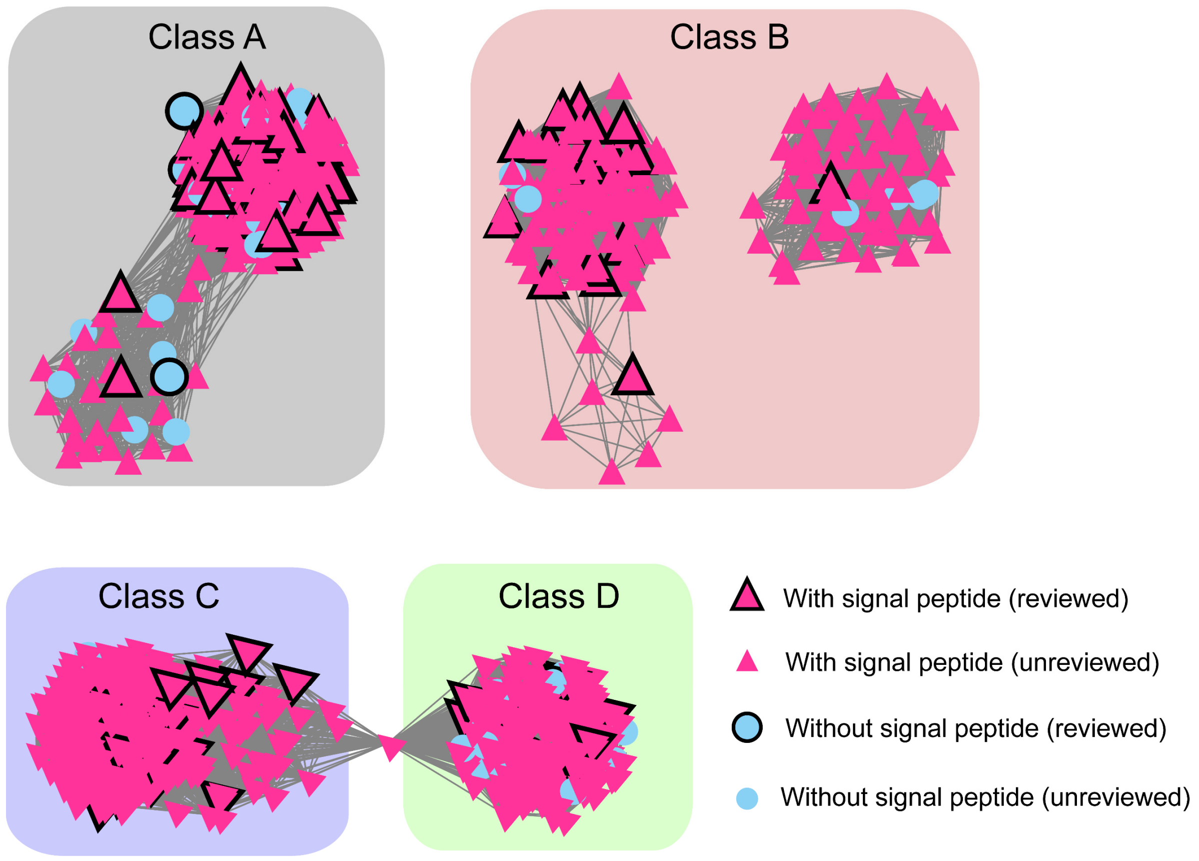

Figure S2. Protein sequence similarity network of β-lactamases annotated by signal peptides. The proteins with signal peptides are shown in pink and triangle form. The protein annotated in Swiss Prot was shown with a black frame.

a

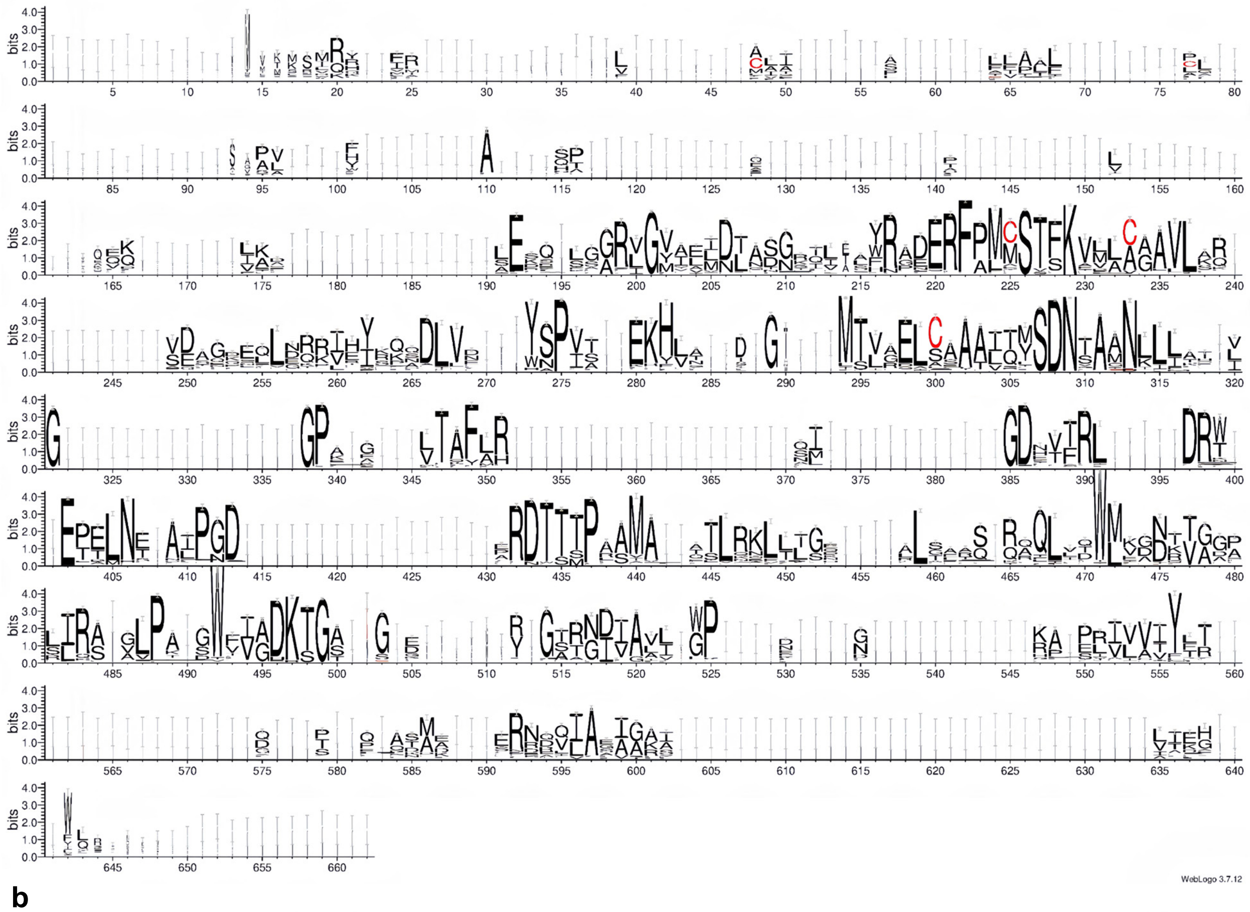

b

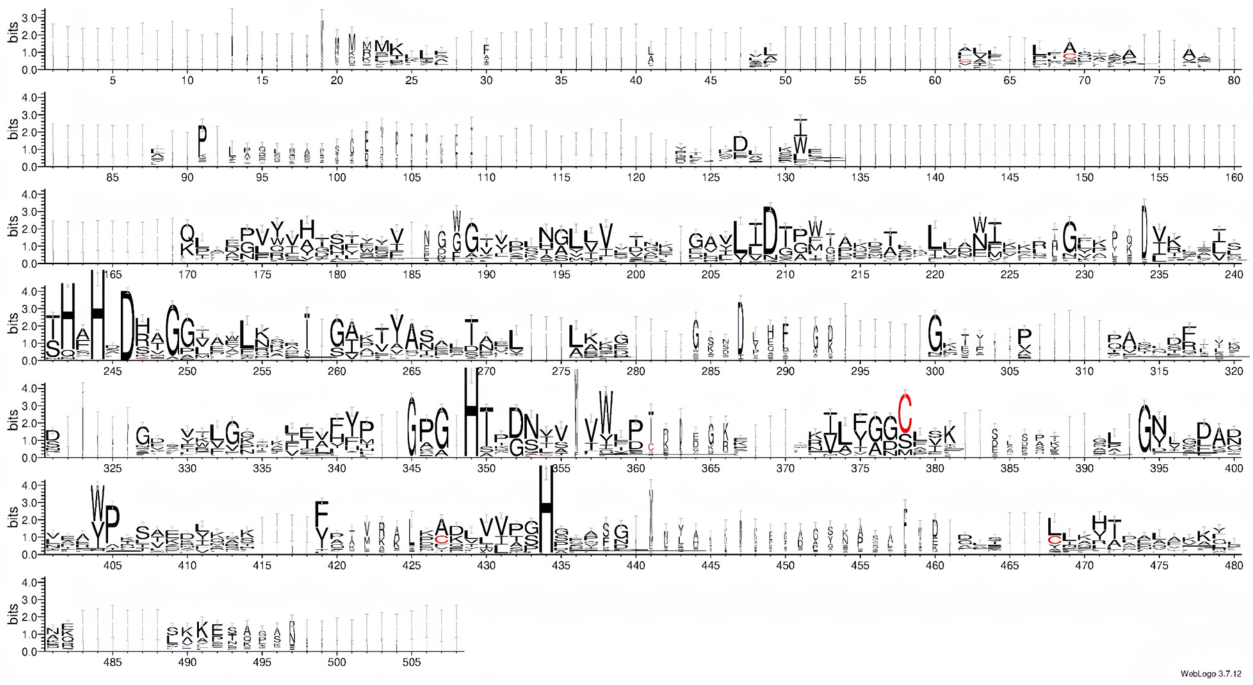

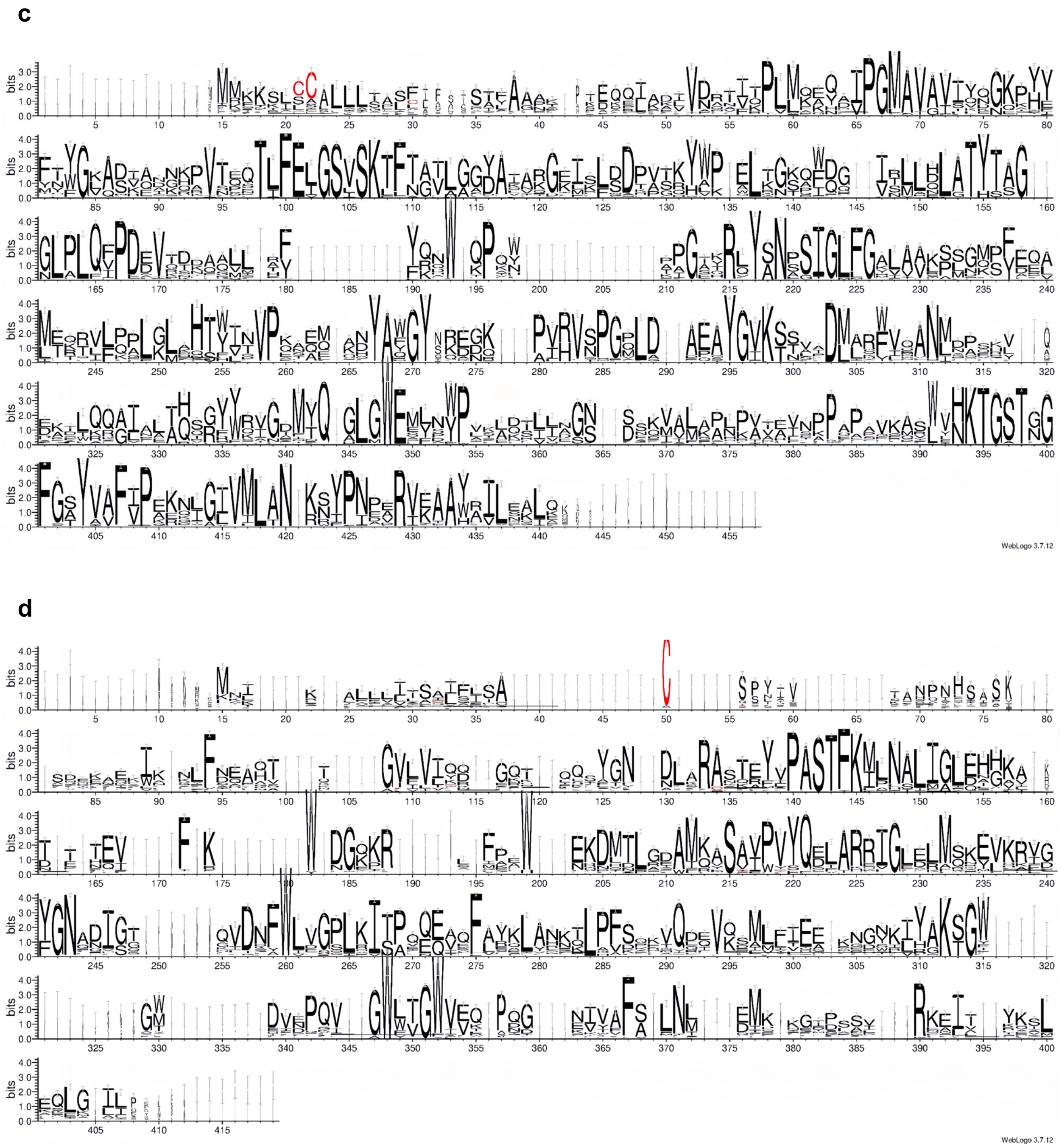

Figure S3. Profile hidden Markov model sequence logos for β-lactamases of class A (a), B (b), C (c), and D (d).

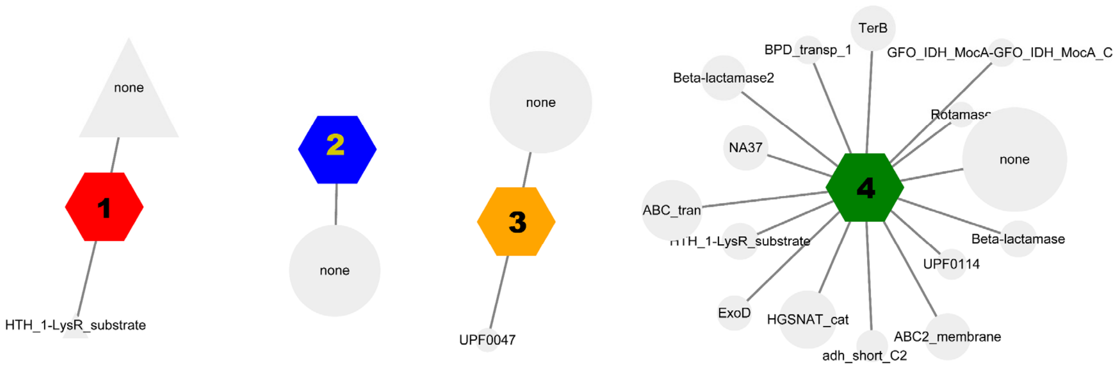

Figure S4. Genome neighborhood networks of Class A β-lactamases. The protein clusters that corresponding to figure 2 are represented by a hexagon. The circles represent the neighboring genes of which edges show genome context relationships with the query cluster. The size of the circle represents the conservation.

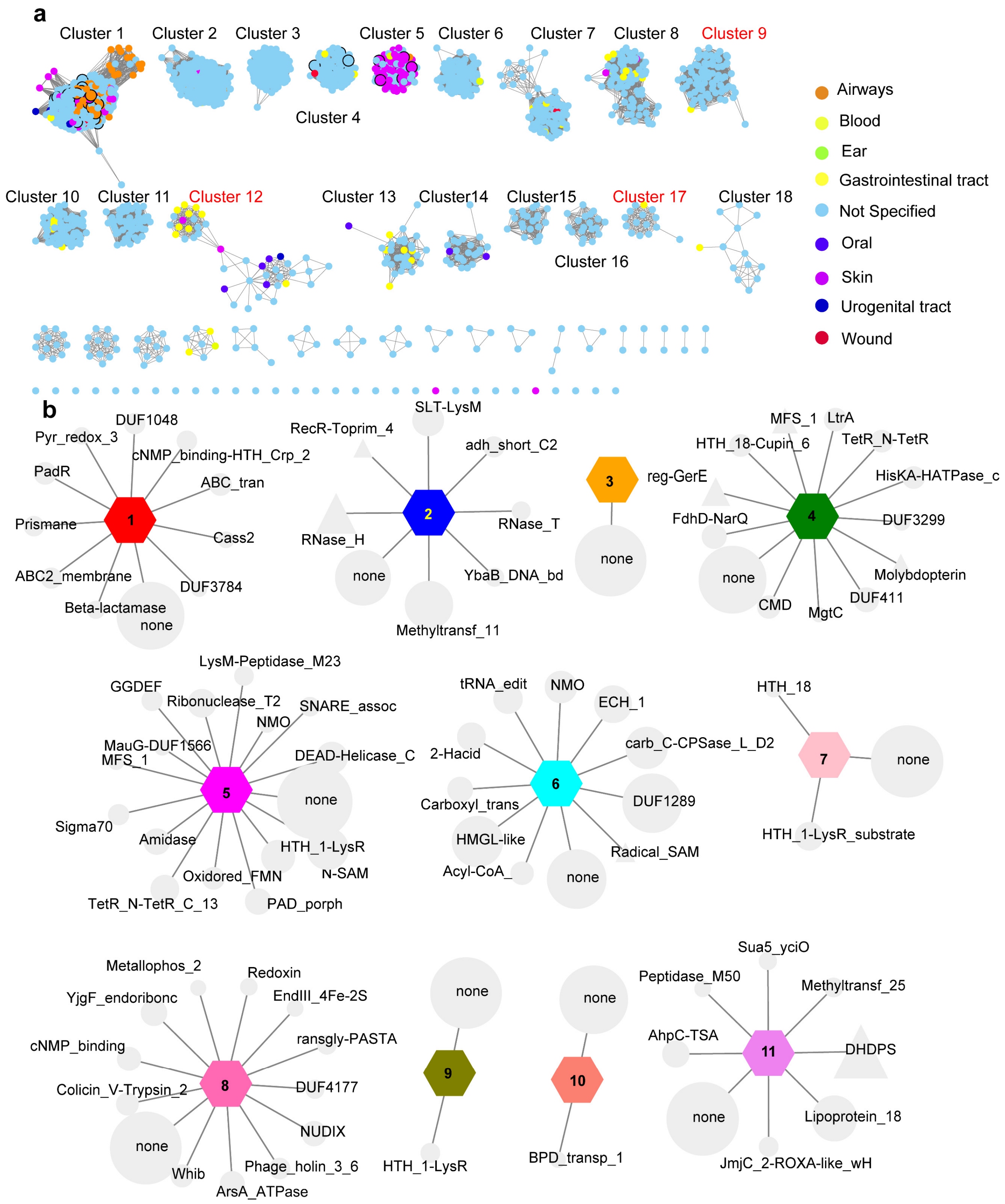
a
Cluster 1
Cluster 2
Cluster 3
Cluster 4
Cluster 5
Cluster 6
Cluster 7
Cluster 8
Cluster 9
Cluster 10
Cluster 11
Cluster 12
Cluster 13
Cluster14
Cluster15
Cluster 16
Cluster 17
Cluster 18
Airways
Blood
Ear
Gastrointestinal tract
Not Specified
Oral
Skin
Urogenital tract
Wound
b
1
DUF1048
Pyr_redox_3
cNMP_binding-HTH_Crp_2
PadR
ABC_tran
Prismane
Cass2
ABC2_membrane
DUF3784
Beta-lactamase
none
2
SLT-LysM
RecR-Toprim_4
adh_short_C2
RNase_T
RNase_H
none
YbaB_DNA_bd
Methyltransf_11
3
reg-GerE
none
4
MFS_1
LtrA
HTH_18-Cupin_6
TetR_N-TetR
HisKA-HATPase_c
FdhD-NarQ
DUF3299
none
Molybdopterin
CMD
MgtC
DUF411
5
LysM-Peptidase_M23
GGDEF
Ribonuclease_T2
SNARE_assoc
NMO
MauG-DUF1566
MFS_1
DEAD-Helicase_C
none
Sigma70
Amidase
HTH_1-LysR
Oxidored_FMN
N-SAM
TetR_N-TetR_C_13
PAD_porph
6
tRNA_edit
NMO
ECH_1
2-Hacid
carb_C-CPSase_L_D2
Carboxyl_trans
DUF1289
HMGL-like
Radical_SAM
Acyl-CoA_
none
7
HTH_18
none
HTH_1-LysR_substrate
8
Metallophos_2
Redoxin
YjgF_endoribonc
EndIII_4Fe-2S
ransgly-PASTA
cNMP_binding
Colicin_V-Trypsin_2
DUF4177
NUDIX
none
Whib
Phage_holin_3_6
ArsA_ATPase
9
none
HTH_1-LysR
10
none
BPD_transp_1
11
Sua5_yciO
Peptidase_M50
Methyltransf_25
AhpC-TSA
DHDPS
none
Lipoprotein_18
JmjC_2-ROXA-like_wH

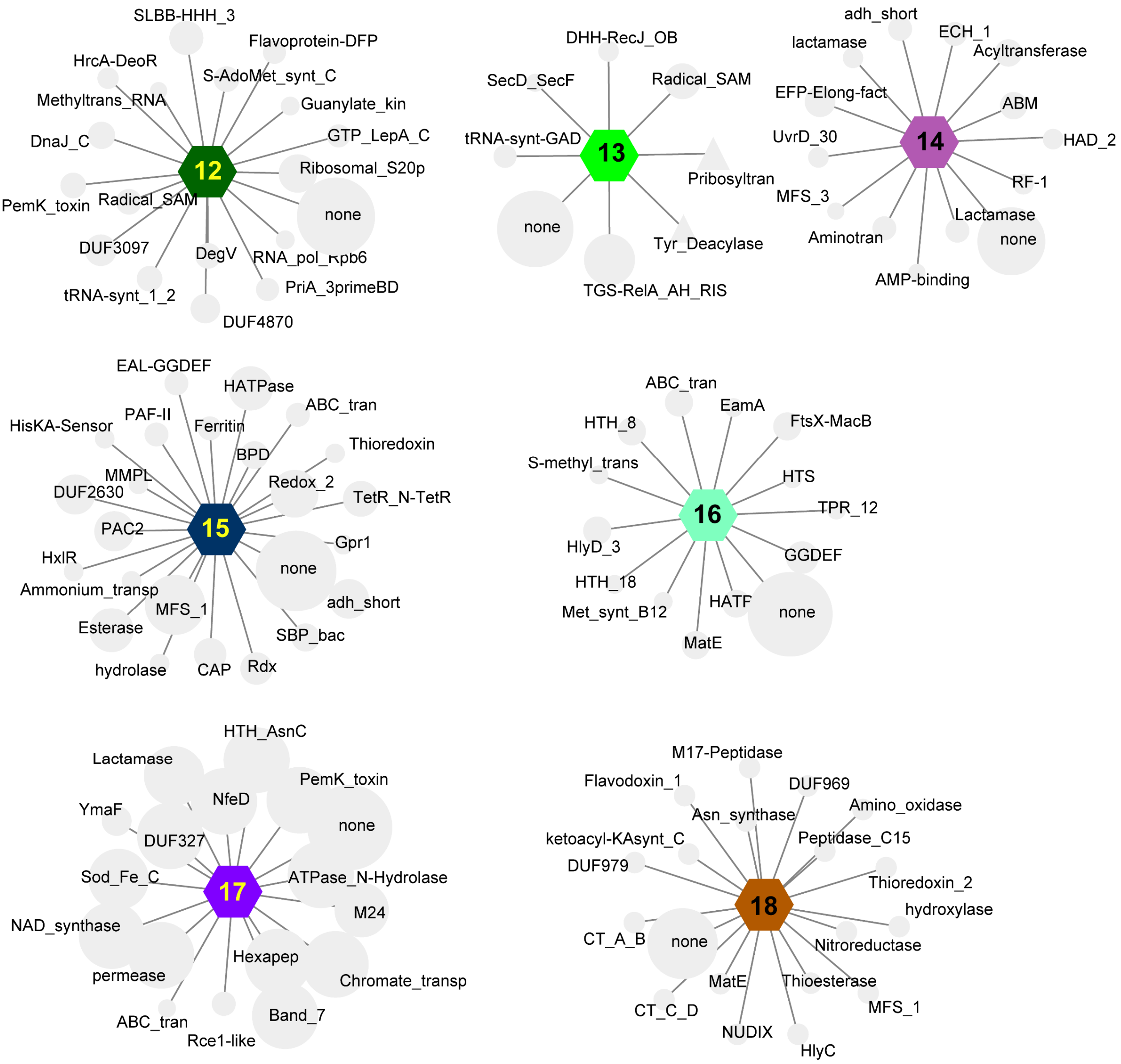

Figure S5. Network analysis of Class B β-lactamases. (a) 2846 homologues of Class B β-lactamases were divided into >18 clusters in the SSN using 30% sequence identity. The homologue proteins were obtained by searching the protein databases using the known proteins identified in fig 2 b. the proteins with the origin of human microbiome of different organs were represented different color and enlarged. The color corresponding the organs were shown in the right part. **(b)** Genome neighborhood networks of Class B β-lactamases. The protein clusters that corresponding to figure a are represented by a hexagon. The circles represent the neighboring genes of which edges show genome context relationships with the query cluster. The size of the circle represents the conservation.

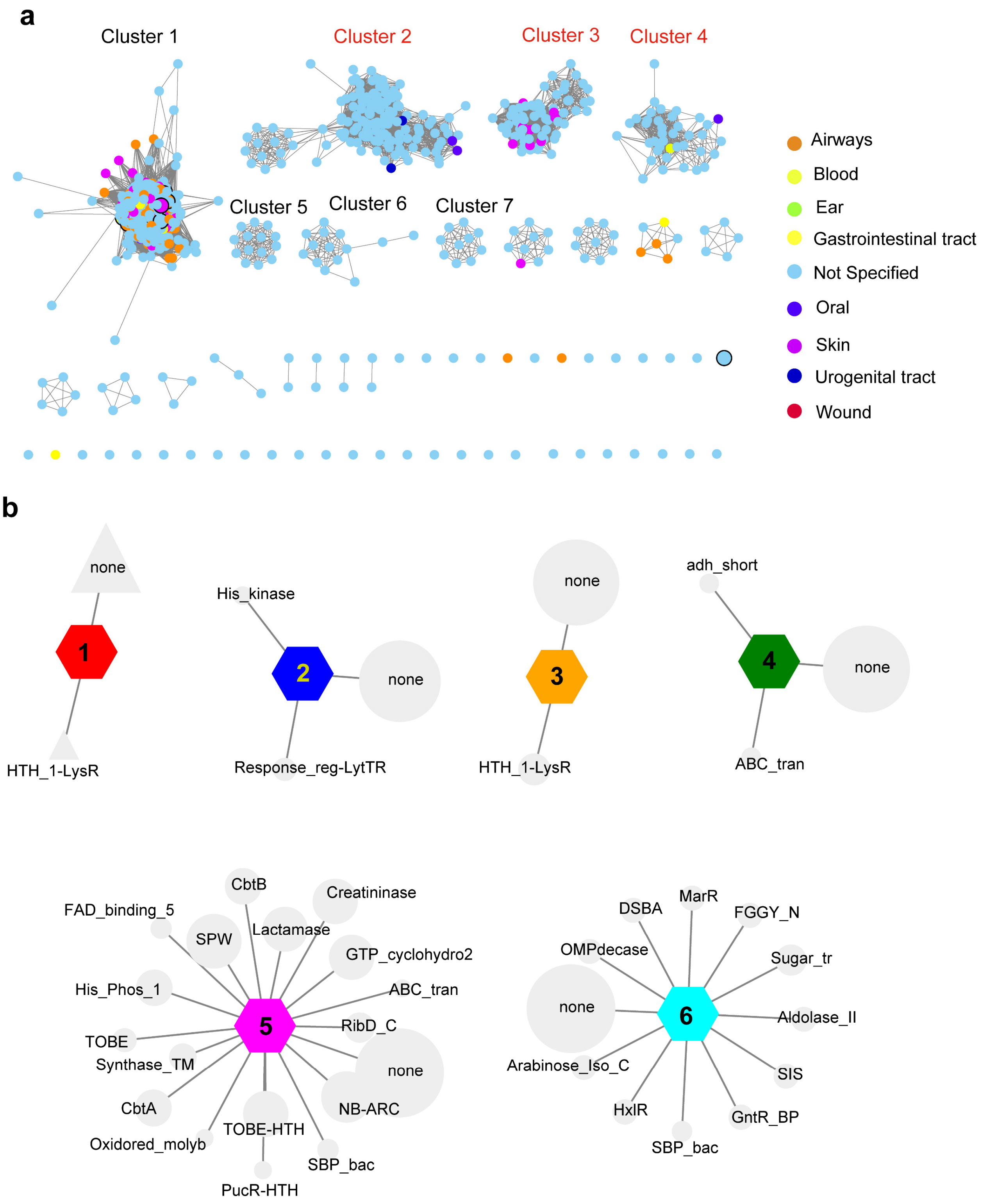

a
Cluster 1
Cluster 2
Cluster 3
Cluster 4
Cluster 5
Cluster 6
Cluster 7
Airways
Blood
Ear
Gastrointestinal tract
Not Specified
Oral
Skin
Urogenital tract
Wound
b
none
1
HTH_1-LysR
His_kinase
2
none
Response_reg-LytTR
none
3
HTH_1-LysR
adh_short
4
none
ABC_tran
CbtB
Creatininase
FAD_binding_5
SPW
Lactamase
GTP_cyclohydro2
His_Phos_1
ABC_tran
5
RibD_C
TOBE
Synthase_TM
none
CbtA
NB-ARC
TOBE-HTH
Oxidored_molyb
SBP_bac
PucR-HTH
MarR
DSBA
FGGY_N
OMPdecase
Sugar_tr
none
6
Aldolase_II
Arabinose_Iso_C
SIS
HxlR
GntR_BP
SBP_bac

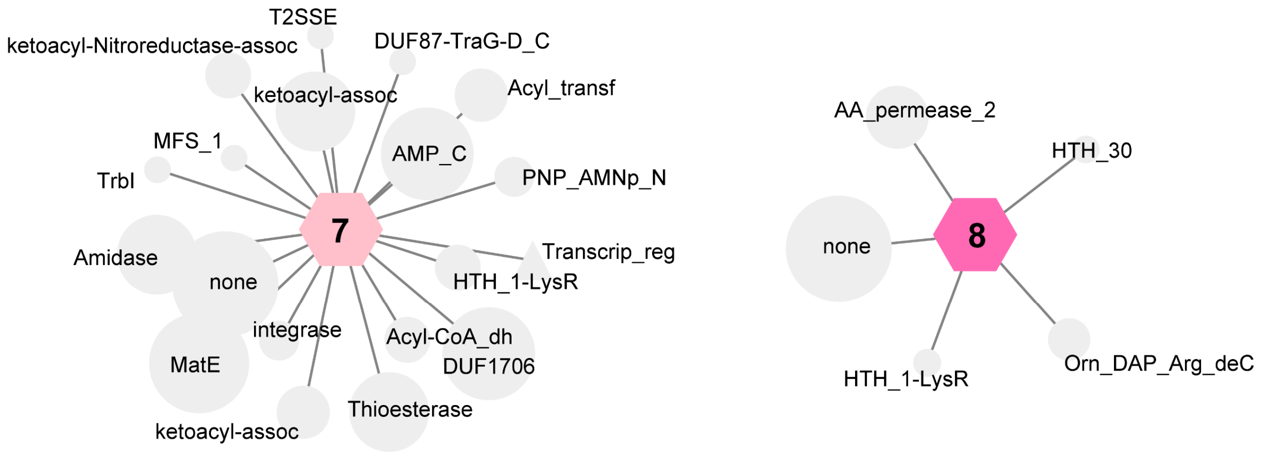

Figure S6. Network analysis of Class c β-lactamases. (a) 4254 homologues of Class C β-lactamases were divided into >8 clusters in the SSN using 30% sequence identity. The homologue proteins were obtained by searching the protein databases using the known proteins identified in fig 2 b. the proteins with the origin of human microbiome of different organs were represented different color and enlarged. The color corresponding the organs were shown in the right part. **(b)** Genome neighborhood networks of Class C β-lactamases. The protein clusters that corresponding to figure a are represented by a hexagon. The circles represent the neighboring genes of which edges show genome context relationships with the query cluster. The size of the circle represents the conservation.

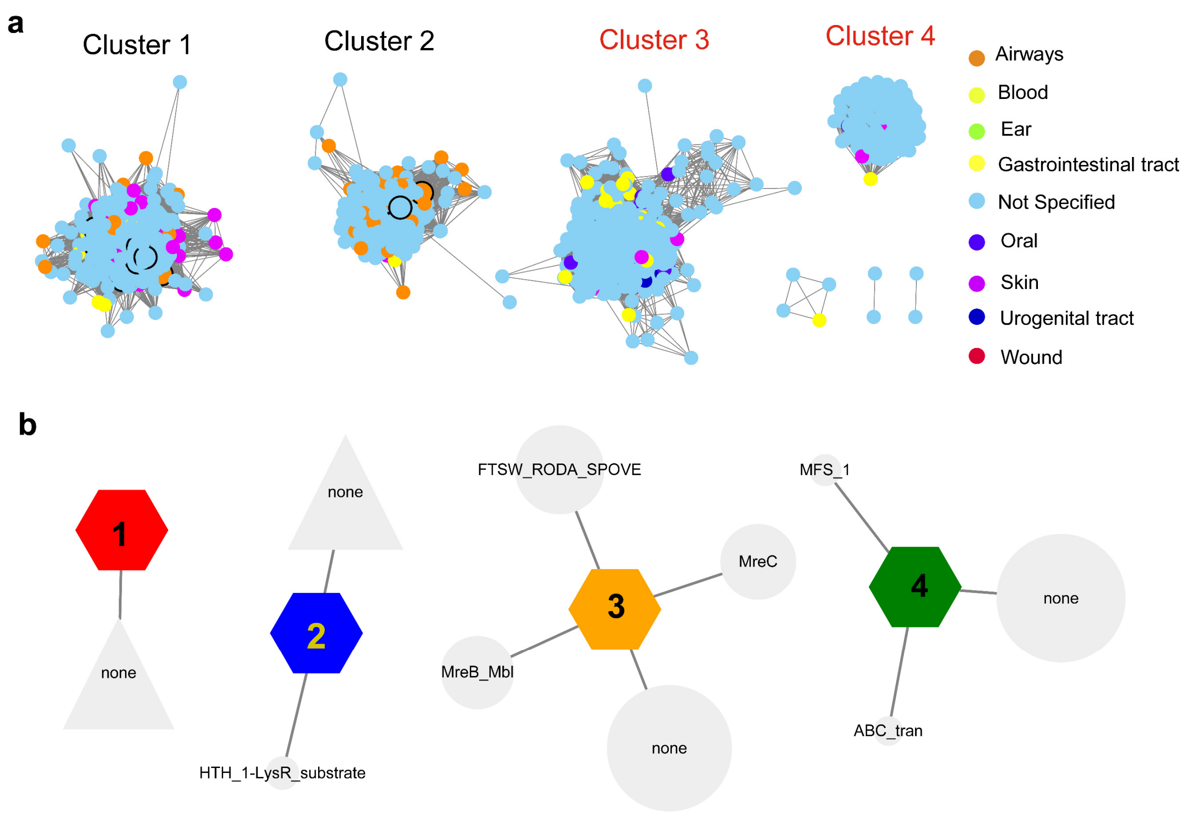

Figure S7. Network analysis of Class D β-lactamases. (a) 3077 homologues of Class D β-lactamases were divided into 4 clusters in the SSN using 30% sequence identity. The homologue proteins were obtained by searching the protein databases using the known proteins identified in fig 2 b. the proteins with the origin of human microbiome of different organs were represented different color and enlarged. The color corresponding the organs were shown in the right part. **(b)** Genome neighborhood networks of Class D β-lactamases. The protein clusters that corresponding to figure a are represented by a hexagon. The circles represent the neighboring genes of which edges show genome context relationships with the query cluster. The size of the circle represents the conservation.

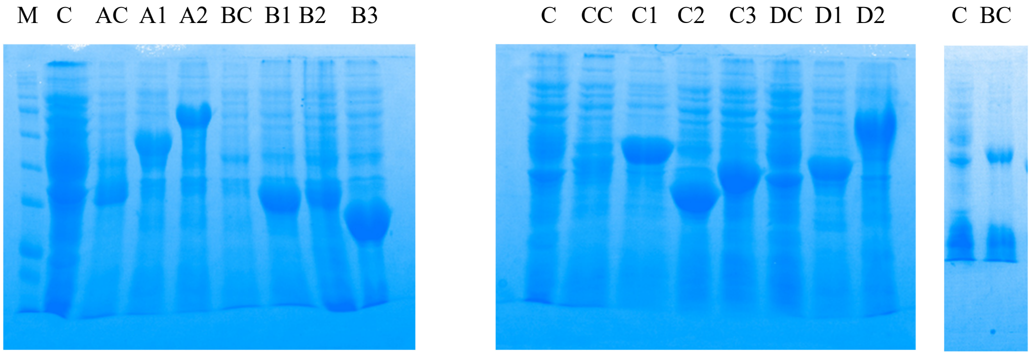

Figure S8. Expression of β-lactamases in *Escherichia coli.* Lane M, protein marker, the molecular mass standards are indicated at the left; Lane C: crude protein extract from *E. coli* cells. Others: crude protein extract from *E. coli* cells with the transformants. The name of the transformants correspond to fig 3. BC was screened twice for expression. The molecular mass observed in the gel differs from the predicted molecular weight, likely due to protein aggregation caused by the presence of signal peptides.

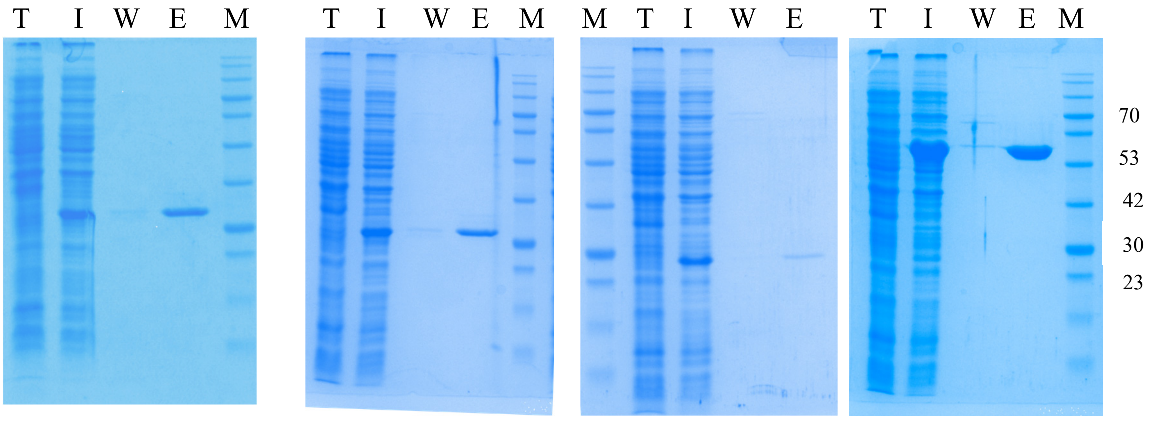

Figure S9. Expression and purification of β-lactamases from *Escherichia coli*. The purification of β-lactamases A1, B1, C1, and D1 are shown in a, b, c, and d. Lane M, protein marker. Lane T, total crude protein extract from non-induced cells; Lane I, crude protein extract from IPTG-induced cells; Lane W, unbound proteins eluted from the Ni-NTA column; Lane E, proteins eluted with 250 mM imidazole.

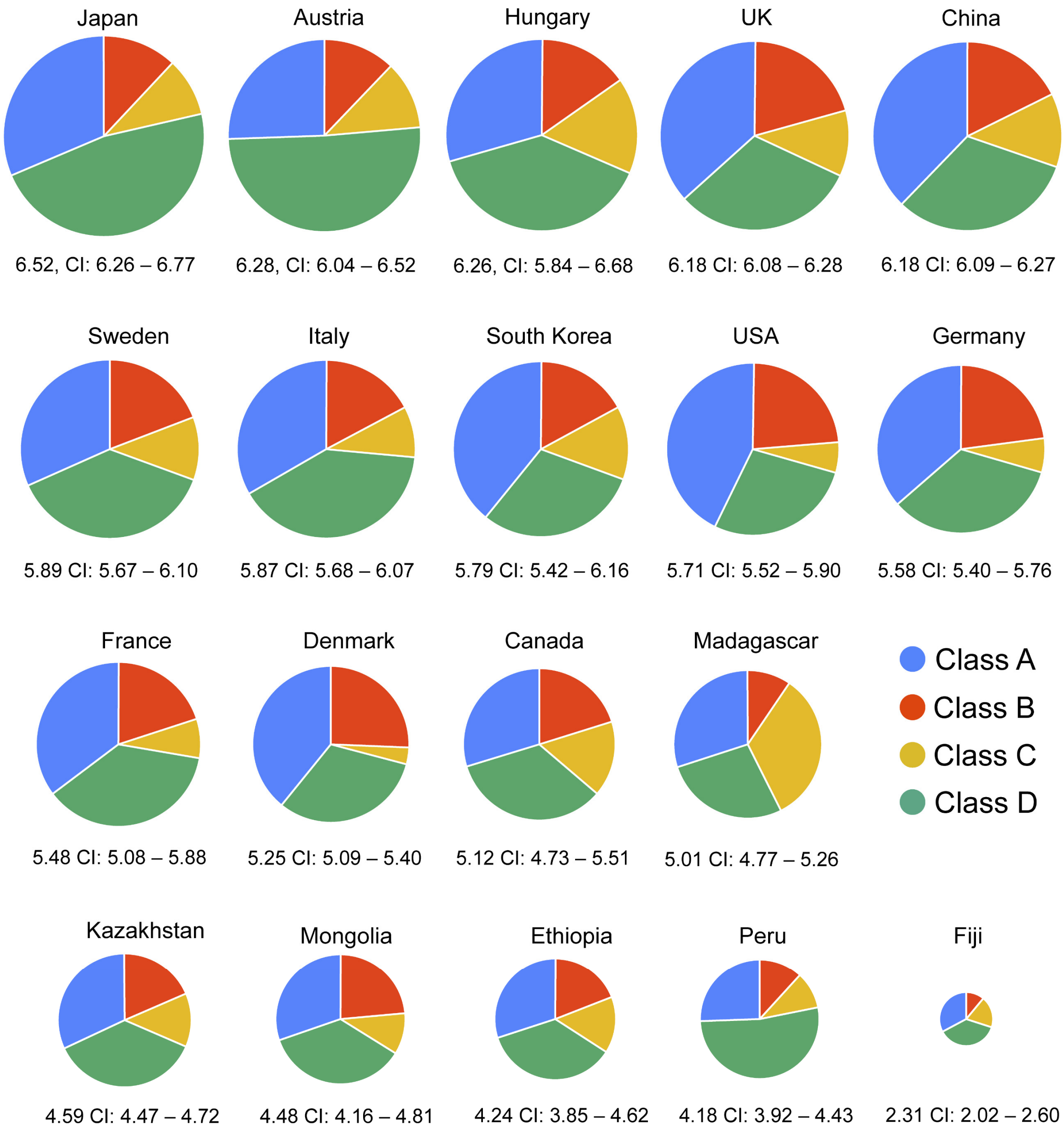

Figure S10. The abundance of the β-lactamases in the gut of healthy participants of 19 countries. The site of the pie shows the average abundance of the β-lactamases in the individuals. The abundance of β-lactamases from the four classes is shown as the indicated color.

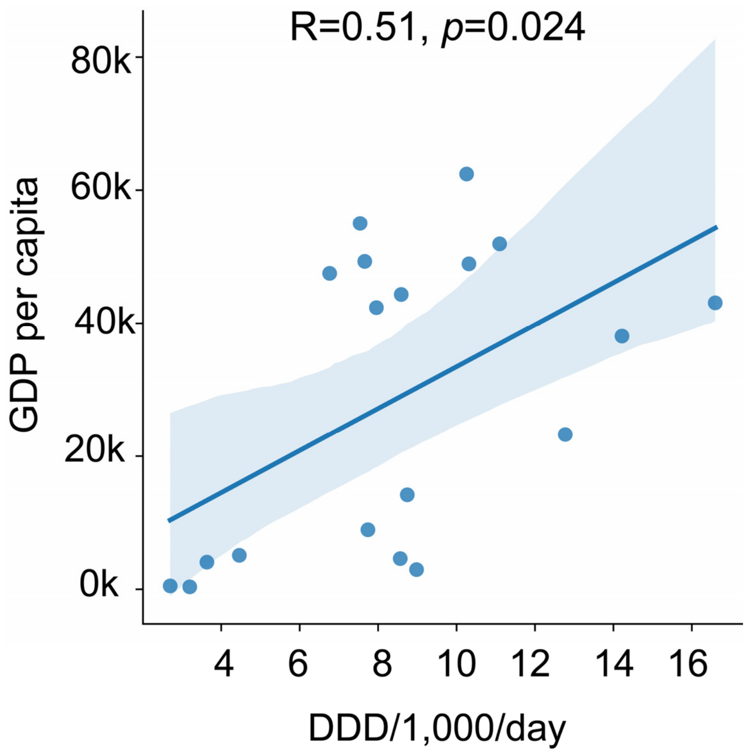

Figure S11. The correlation between total antibiotic consumption rates estimated from defined daily doses (DDD) per 1000 population per day and the income (Gross domestic product (GDP) per captia) of the 19 countries from 2000-2018.

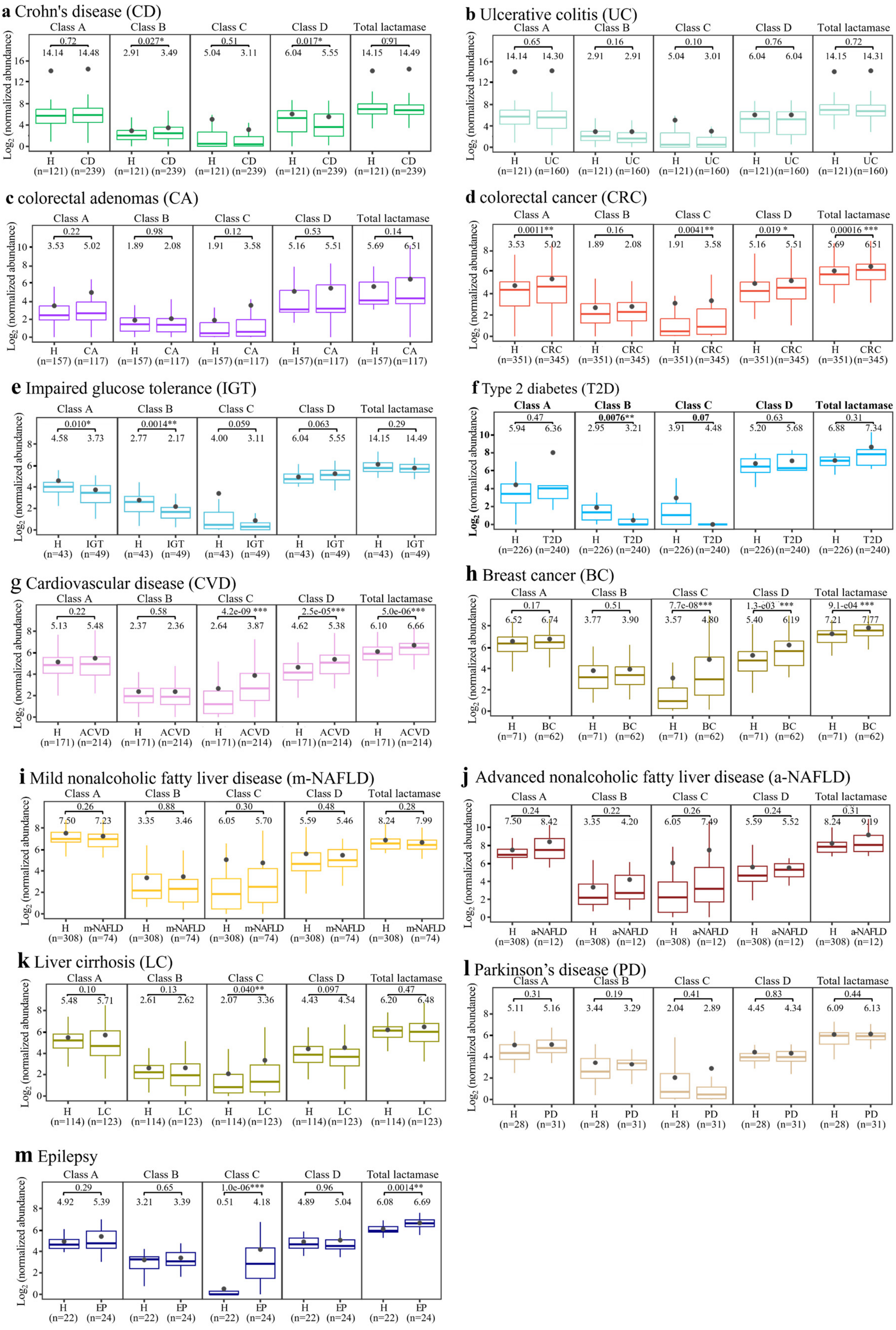
a Crohn's disease (CD)
b Ulcerative colitis (UC)
c colorectal adenomas (CA)
d colorectal cancer (CRC)
e Impaired glucose tolerance (IGT)
f Type 2 diabetes (T2D)
g Cardiovascular disease (CVD)
h Breast cancer (BC)
i Mild nonalcoholic fatty liver disease (m-NAFLD)
j Advanced nonalcoholic fatty liver disease (a-NAFLD)
k Liver cirrhosis (LC)
l Parkinson's disease (PD)
m Epilepsy
Class A
Class B
Class C
Class D
Total lactamase
Log2 (normalized abundance)

Figure S12. Abundance of *β-lactamase* genes in metagenomic data from controls and individuals with diseases. The sample number of controls and diseases are shown below the box plot. The abbreviations of the diseases are: **(a)** UC: ulcerative colitis, **(b)** CD: Crohn's disease, **(c)** CA: colorectal adenomas, **(d)** CRC: colorectal cancer, **(e)** IGT: Impaired glucose tolerance, **(f)** T2D: Type 2 diabetes, **(g)** CVD: cardiovascular disease, **(h)** BC: Breast cancer, **(i)** mild NAFLD: non-alcoholic fatty liver disease, **(g)** advanced NAFLD: advanced non-alcoholic fatty liver disease, **(k)** LC: liver cirrhosis, **(l)** PD: Parkinson's disease, **(m) e**pilepsy. The number above the plot shows the generalized fold change. The significant value is determined using which is determined using two-sample Wilcoxon rank sum test or two-sample Student's t-test (shown as t after the significant value) as described in Methods. The cohort's information was in Table S3.

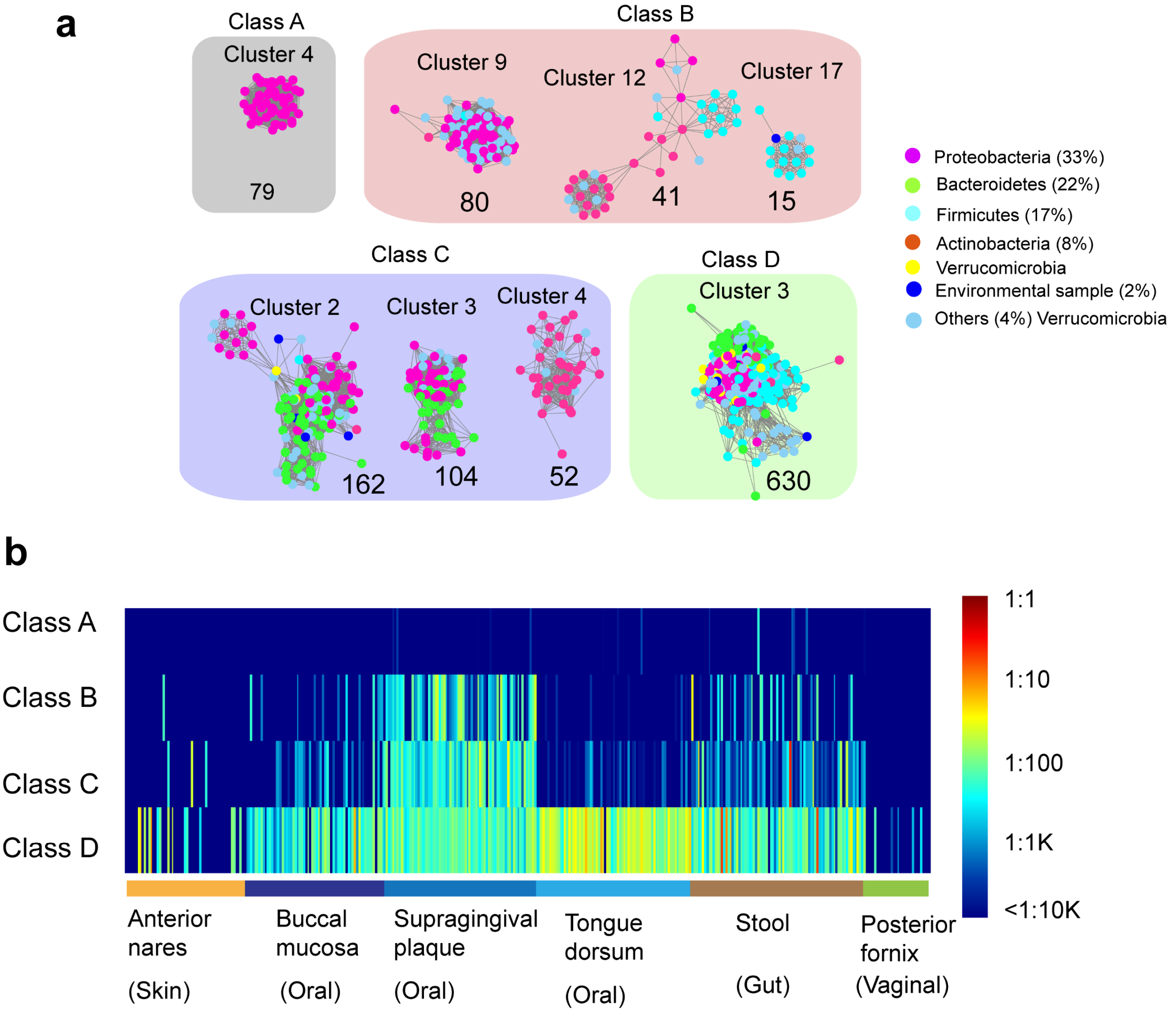

Figure S13. Summary of the newfound β-lactamase. (a) protein sequence similarity network of the newfound β-lactamases using 40% cutoff of sequence identity. Proteins from the same phylum are painted by the same color and the percentage from the phylum is listed at the right part. **(b)** Heatmap of the abundance and distribution of the four class β-lactamase across six body sites, as determined through ShortBRED analysis.

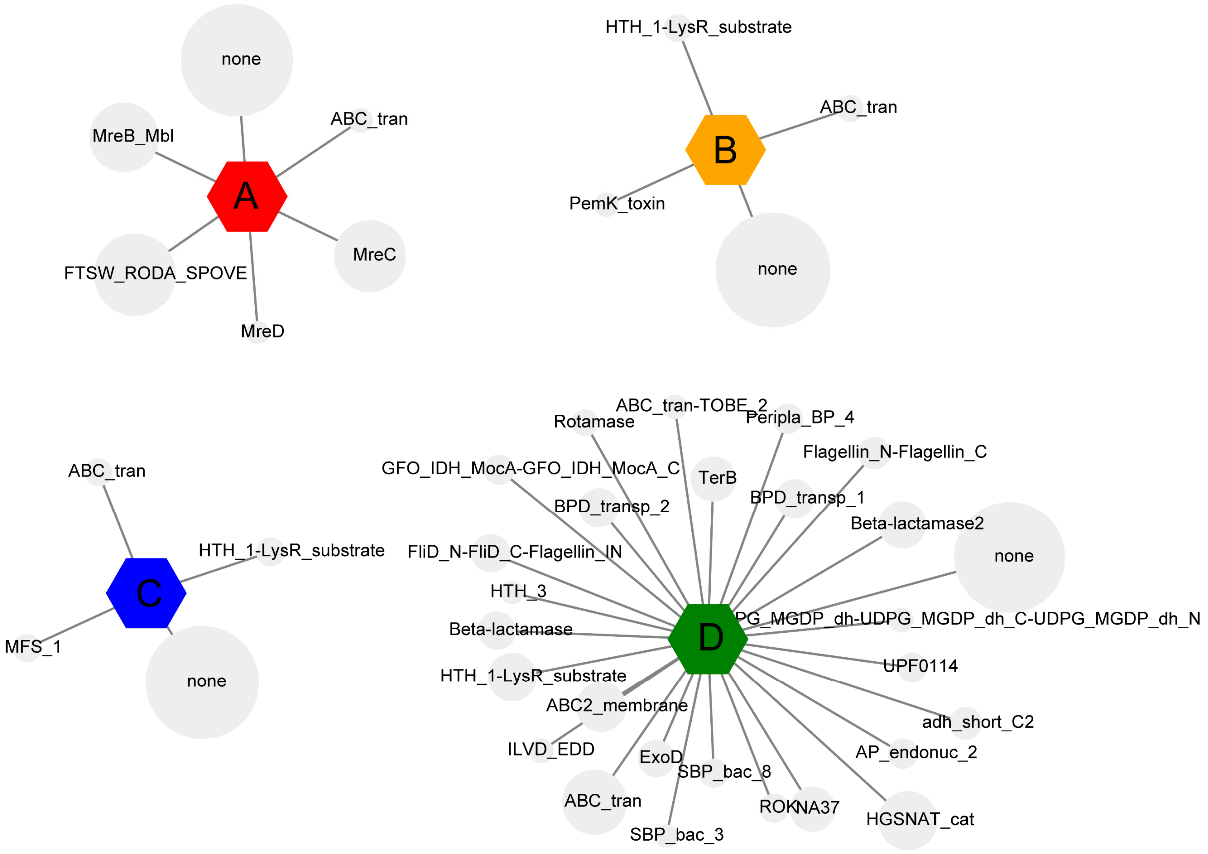

Figure S14. Genome neighborhood networks of newfound β-lactamases belonging to the four classes. The protein clusters that corresponding to figure a are represented by a hexagon. The circles represent the neighboring genes of which edges show genome context relationships with the query cluster. The size of the circle represents the conservation.

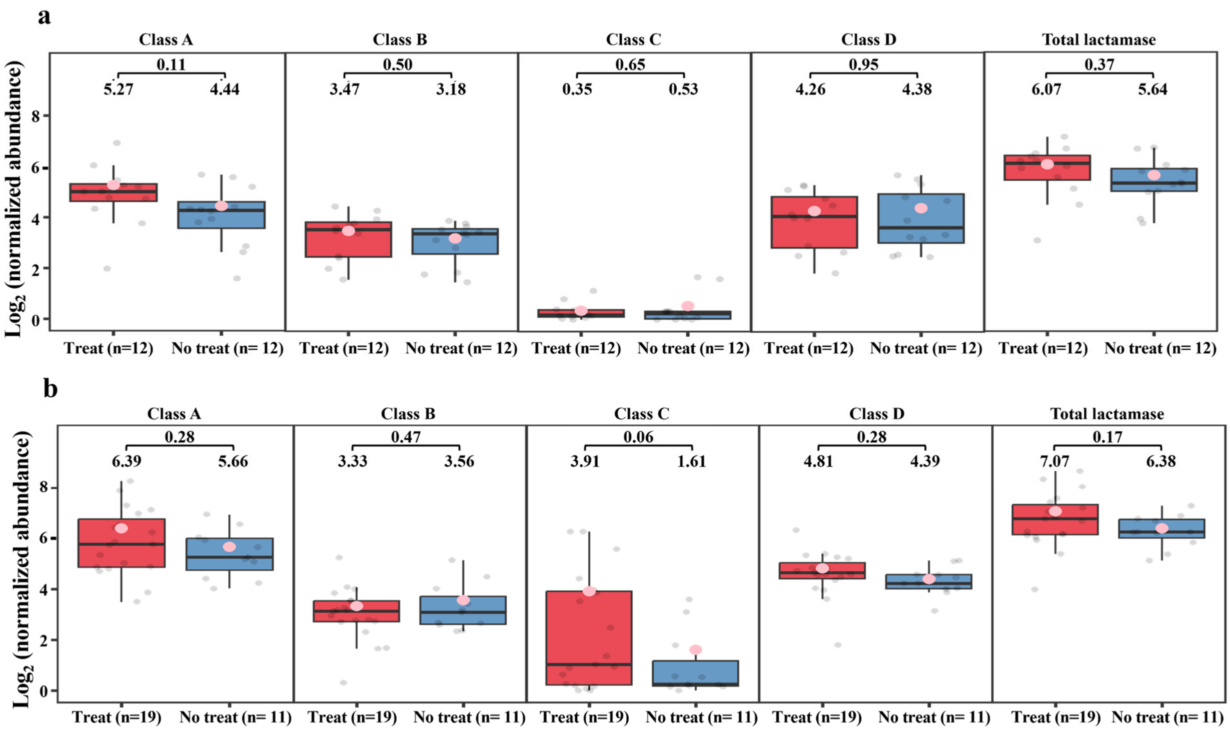

Figure S15. Abundance of *β-lactamase* genes in metagenomic data from controls (No treat) and individuals with antibiotics (Treat). (a) The cohort (PRJEB20800) from Denmark was analyzed to compare the difference between healthy individuals who had taken meropenem, vancomycin, or gentamicin and a control group who had not taken any antibiotics. **(b)** The cohort (PRJEB38625; PRJEB42013) from Spain and Germany was analyzed to compare the difference between patients with pancreatitis who had taken antibiotics and a control group whose antibiotic use is unknown. The detail cohort's information was in Table S4.
